# Hypoxia-driven transcriptional activation of MIR100HG by HIF-1α contributes to adaptive gene regulation in hepatocellular carcinoma

**DOI:** 10.1007/s10142-026-01899-9

**Published:** 2026-06-15

**Authors:** Nelin Hacioglu, Aylin Turkoglu Dulger, Sevin Avsar Koc, Esra Tokay, Meltem Alper, Feray Kockar

**Affiliations:** 1 Faculty of Science and Literature, Department of Molecular Biology and Genetics, Balikesir University, Balikesir, Turkey; 2 Institute of Oncology, Department of Translational Oncology, Dokuz Eylul University, Izmir, Turkey

**Keywords:** MIR100HG, LncRNA, Hepatocellular cancer, Hypoxia, RNA-seq, Hep3B, SNU-398

## Abstract

Background: Hepatocellular carcinoma (HCC) develops in a chronically hypoxic microenvironment; however, the contribution of hypoxia-regulated long non-coding RNAs (lncRNAs) remains poorly defined. Aim: We aimed to identify MIR100HG as a hypoxia-responsive regulator in hepatocellular carcinoma (HCC) and to define its transcriptional control by hypoxia-inducible factor 1 alpha. Methods: Basal MIR100HG expression was analyzed in four hepatocellular carcinoma cell lines (Hep3B, Huh-7, SNU-398, and SNU-475) and non-tumorigenic Clone-9 hepatocytes. Hypoxia was induced by cobalt (II) chloride. Proliferation, migration, and clonogenicity were evaluated using functional assays. Promoter analysis, electrophoretic mobility shift assay, chromatin immunoprecipitation quantitative polymerase chain reaction, and 5′-deletion luciferase assays were used to map the hypoxia response elements. Transcriptomic profiling was performed using RNA sequencing, followed by gene set enrichment analysis. Gain- and loss-of-function experiments validated the downstream effects. Results: MIR100HG expression was highest in Hep3B cells and was significantly induced under hypoxia in all hepatic models, indicating a conserved, oxygen-sensitive response. Hypoxia suppressed proliferation, migration, and clonogenicity, with the strongest inhibition observed in non-tumorigenic hepatocytes under hypoxic conditions. Functional hypoxia response elements were identified within the − 815/+231 promoter region, demonstrating direct transcriptional activation by hypoxia-inducible factor 1 alpha. Transcriptomic analyses revealed the enrichment of hypoxia signaling, oxidative stress, proteasome activity, RNA metabolism, autophagy, and ferroptosis pathways. While hypoxia induced broad transcriptional reprogramming, MIR100HG modulated the expression of selected hypoxia-responsive genes, including the PAGE1 and CTAG2, as demonstrated under hypoxic conditions by RNA-seq and functional perturbation experiments. Conclusions: MIR100HG is a hypoxia-inducible factor 1 alpha-driven long non-coding RNA associated with hypoxia-responsive transcriptional programs in hepatocellular carcinoma and represents a potential biomarker and therapeutic target.

**Supplementary Information:**

The online version contains supplementary material available at 10.1007/s10142-026-01899-9.

## Introduction

Long non-coding RNAs (lncRNAs) are defined as RNA molecules longer than 200 nucleotides that do not have protein-coding capacity (Shang et al. 2016). Recent discoveries have revealed that lncRNAs play important roles in various cellular processes, such as transcription, post-transcription, and epigenetic gene regulation (Farooqi et al. 2018; Peng et al. 2019). They play an essential role in cell proliferation, differentiation, metastasis, and apoptosis. In cancer biology, increased expression of lncRNAs has been associated with many types of cancer (Bagheri et al. 2019).

MIR100HG is a lncRNA located in the 11q24.1 chromosomal region of the human genome. MIR100HG serves as the host gene for the miR-100, let-7a-2, and miR-125b-1/miR-125b-5p microRNA (miRNA) cluster (Lu et al., 2017). MIR100HG has also been implicated in the regulation of cell cycle progression (Braga et al. 2023). The expression of this lncRNA can be dysregulated in different cancer types, where it may function as either an oncogene or a tumor suppressor, depending on the tumor type (Balas and Johnson 2018). In the literature, MIR100HG has been shown to function as a tumor promoter in human cancers, including neuroblastoma, leukemia, breast cancer, colorectal cancer, bladder cancer, cervical cancer, osteosarcoma, pancreatic ductal adenocarcinoma, and head and neck squamous cell carcinoma, through bioinformatics and expression studies (Wu et al. 2022). Although hypoxia is well recognized as a key determinant of hepatocellular carcinoma (HCC) biology, its potential regulation of MIR100HG has not been adequately explored. MIR100HG has been shown to be generally upregulated in HCC tissues, indicating poor prognosis and advanced tumor stages. For example, high MIR100HG expression has been shown to increase with increased cell proliferation and invasion capacity in HCC, suggesting an oncogenic role (Wang et al. 2019). The expression of MIR100HG is significantly higher in HCC tissues than in adjacent tumor tissues, suggesting a potential role as an oncogene in this malignancy (Li et al., 2021). The researchers conducted expression analyses using patient tissues and various HCC cell lines, including Hep3B, HepG2, SK-HEP1, and Huh7 cell lines. Subsequently, they performed gene silencing studies using siRNA in two selected cell lines and validated their findings using MTT, scratch, and invasion assays. Subsequently, bioinformatics analysis identified one miR-146b-5p) and one associated gene (CBX6) (Li et al., 2021).

Hypoxia plays a significant role in HCC development and progression. Since HCC typically arises in the setting of cirrhosis induced by chronic liver injury, fibrinogenesis resulting from liver injury and cirrhosis leads to a reduction in vascularization, which contributes to hypoxia (Affo et al. 2016; Chen et al. 2023). HCC may present as cavitary lesions in the liver due to rapid tumor growth, leading to necrosis and hypoxia (Lin and Wu 2015).

Despite accumulating evidence linking MIR100HG to cancer progression, its regulation in hypoxic hepatocellular carcinoma, a setting where hypoxia is a dominant microenvironmental driver, remains unexplored. Given the central role of HIF-1α in orchestrating hypoxia-responsive transcriptional programs, understanding whether MIR100HG is an oxygen-sensitive lncRNA and how it contributes to hypoxia-driven cellular remodeling is critical. In this study, we systematically investigated the expression, transcriptional regulation, and functional impact of MIR100HG under chemically mimicked hypoxic conditions in five liver-derived cell models. We demonstrate that MIR100HG is robustly induced by hypoxia in all hepatic cell lines, identify direct and specific HIF-1α binding to core hypoxia-response elements within its promoter through EMSA, luciferase deletion mapping, and ChIP-qPCR, and reveal that MIR100HG governs key oxygen-responsive transcriptional networks as shown by RNA-seq and GSEA. Functional assays further confirmed that hypoxia suppresses cell proliferation, migration, and clonogenicity while simultaneously elevating MIR100HG expression, suggesting an adaptive role rather than a purely oncogenic role. In.

In addition, we investigated the hypothesis that MIR100HG acts as a central regulatory node integrating hypoxic signaling with RNA metabolic control, proteostasis, and immune-related transcriptional outputs. By combining RNA-seq-based transcriptomic profiling with targeted qRT-PCR validation and functional gain- and loss-of-function approaches, we demonstrated that MIR100HG orchestrates a multidimensional hypoxic gene network, prominently involving cancer/testis antigens (CTAG2 and PAGE1) and stress–inflammation–associated genes (OAS2, IL1RN, CXCL10, and GBP1). Our findings identify MIR100HG as a previously unrecognized and versatile regulator of hypoxic adaptation and transcriptional plasticity in cancer cells, underscoring its potential relevance in tumor microenvironment remodeling, immune evasion, and stress-responsive oncogenic programs.

## Materials and methods

### Cell culturing

Two epithelial Hep3B (RRID: CVCL_C8Q9) and Huh-7 (RRID: CVCL_0336) and two mesenchymal-like SNU-398(RRID: CVCL_0077) and SNU-475 (RRID: CVCL_0497) hepatocellular carcinoma cells, and one healthy rat epithelial liver Clone-9 (RRID: CVCL_E2S6) cell used in the study were obtained from ATCC. Cells were cultured in Dulbecco’s Modified Eagle Medium (DMEM, high glucose; Gibco, Thermo Fisher Scientific, Cat#11965-092) supplemented with 10% fetal bovine serum (FBS; Gibco, Cat#10270-106) and 1% penicillin-streptomycin (Gibco, Cat#15140-122). Cells were maintained at 37 °C in a humidified incubator with 5% CO₂.

### Cloning of MIR100HG promoter constructs

The upstream promoter region of the human MIR100HG gene was analyzed to identify putative transcription factor binding sites and regulatory elements. The 1013 bp sequence upstream of the MIR100HG transcription start site (GenBank accession no: NR_024430.2) was retrieved and analyzed using the PROMO web tool (Version 3.0.2; based on TRANSFAC v8.3) to predict transcription factor-binding sites. CpG island prediction was performed using the CpG Plot tool (EMBOSS). Primers were designed to amplify full-length and truncated fragments of the MIR100HG promoter region. Primer sequences were optimized based on melting temperature (Tm), GC content, and specificity was verified using NCBI BLAST. To facilitate directional cloning into the pMetLuc luciferase reporter vector, HindIII and XhoI recognition sites were added to the 5’ ends of the forward and reverse primers, respectively. Primer sequences are provided in Supp. Table 1.

The 1244 bp upstream region of the MIR100HG gene was amplified by PCR using Hep3B genomic DNA as a template and cloned into the pMetLuc vector between the HindIII and XhoI sites, resulting in the pMeT_MIR100HG [−1013/+231] construct (Tokay and Kockar 2016b). To evaluate the functional activity of the distinct promoter regions, four 5-truncated fragments were generated using the same strategy: pMeT_MIR100HG [−815/+231], pMeT_MIR100HG [−678/+231], pMeT_MIR100HG [−397/+231], and pMeT_MIR100HG [−188/+231]. All constructs were verified by restriction enzyme digestion and Sanger sequencing (BM Labosis, Ankara, Turkey).

### Cell viability and cytotoxicity assay

MTT (3-[4,5-dimethylthiazol-2-yl]−2,5-diphenyltetrazolium bromide) colorimetric method was applied for cell proliferation, viability and cytotoxicity assessment (Hacıoğlu et al. 2021). Cells were seeded in 96-well plates at 2 × 10⁴ cells/well and maintained overnight in a 37 °C incubator containing 5% CO₂. The next day, 300 µM CoCl₂ (CoCl₂; Sigma-Aldrich, Cat#C8661) was added to the cells, and the cytotoxic effects were evaluated. At 24, 48, and 72 h after application, MTT solution was added to each well at a final concentration of 0.5 mg/mL, and the cells were incubated for 4 h. After incubation, the medium was removed, and the formed formazan crystals were dissolved in isopropanol containing 0.004 M HCl. The absorbance values were measured spectrophotometrically at 550 nm. Cell viability was expressed as a percentage relative to untreated control cells. All experiments were performed in triplicate, and data were analyzed using GraphPad Prism software (GraphPad Software, USA).

### qRT-PCR

Total RNA was isolated from HUVECs using the GeneJET RNA Purification Kit (Thermo Fisher Scientific, Cat#K0732) according to the manufacturer’s instructions. Briefly, cells were harvested by trypsinization, pelleted by centrifugation at 1,000 rpm for 5 min, washed with phosphate-buffered saline (PBS), and centrifuged again prior to RNA extraction. RNA concentration and purity were assessed using a Multiskan™ GO microplate spectrophotometer with µDrop™ Plate (Thermo Fisher Scientific) by measuring absorbance at 260 and 280 nm. RNA samples with acceptable purity (A260/A280 ratio of 1.8–2.0) were used for further analysis and stored at − 80 °C (Tokay and Köçkar, 2016a). First-strand cDNA was synthesized from total RNA using a commercially available cDNA synthesis kit (Thermo Fisher Scientific) according to the manufacturer’s protocol. Quantitative real-time PCR (qRT-PCR) was performed in a total reaction volume of 10 µL containing 5 µL SYBR Green Master Mix (Amplicon, Denmark), 1 µL cDNA template, 0.5 µL of each forward and reverse primer and nuclease-free water. The primer sequences used are listed in Supp. Table 1. Amplification was carried out using a LightCycler 480 system (Roche Diagnostics, Germany) under the following conditions: initial denaturation at 95 °C for 10 min, followed by 35 cycles of 95 °C for 15 s, 58–60 °C for 30 s, and 72 °C for 30 s. Melting curve analysis was performed to confirm amplification specificity. Data were analyzed by Livak (2^–ΔCt) method (Livak and Schmittgen 2001). For each sample, the Ct value of the relevant gene was normalized to the Ct value of human β2-microglobulin (β2M), which was used as an internal control (Hacıoğlu et al., 2020; Tokay et al. 2021).

### Western blot

Western blot analysis was performed to confirm hypoxia induction at the protein level in Hep3B, SNU-398, and Clone-9 cell lines by assessing HIF-1α expression. Cells were cultured in 100-mm dishes under normoxic conditions and treated with 300 µM cobalt chloride (CoCl₂; Sigma-Aldrich, Cat#C8661) to induce chemical hypoxia. Following treatment, cells were washed with cold phosphate-buffered saline (PBS) and lysed using RIPA buffer (Thermo Fisher Scientific, Cat#89900) supplemented with protease and phosphatase inhibitor cocktails (Sigma-Aldrich, Cat#P8340 and Cat#P5726). Lysates were clarified by centrifugation at 12,000 × g for 15 min at 4 °C. Protein concentrations were determined using the Bradford assay, and equal amounts of protein (30–50 µg) were separated on 10% SDS-PAGE gels and transferred onto PVDF membranes (Millipore, Cat#IPVH00010). Membranes were blocked with 5% non-fat dry milk in TBST for 1 h at room temperature and incubated overnight at 4 °C with primary antibodies against HIF-1α (Cell Signaling, Cat# 36169) and β-actin (loading control; [Sigma Aldrich, Cat# A3854]). After washing, membranes were incubated with HRP-conjugated secondary antibody for 1 h at room temperature. Protein bands were visualized using an enhanced chemiluminescence (ECL) detection system (Thermo Fisher Scientific, Cat#32106) and imaged using a chemiluminescence detection system. Densitometric analysis was performed using ImageJ software (NIH, USA) (Alper et al. 2025; Altuntaş et al. 2023; Tokay 2021).

### Electrophoretic mobility shift analysis (EMSA)

To evaluate the functional binding of HIF-1α to its cognate DNA elements under hypoxic conditions, electrophoretic mobility shift assay (EMSA) was performed. Hep3B cells were seeded in 100-mm dishes at a density of 3 × 10⁶ cells/dish and treated with 300 µM cobalt chloride (CoCl₂; Sigma-Aldrich, Cat#C8661) to induce chemical hypoxia. Nuclear extracts were prepared 6 h post-treatment. Cells were washed with cold phosphate-buffered saline (PBS) and resuspended in TEN buffer (10 mM Tris-HCl, 1 mM EDTA, 100 mM NaCl), followed by centrifugation at 13,000 × g for 1 min at 4 °C. Pellets were subsequently lysed using buffer A and incubated in high-salt buffer C to obtain nuclear extracts, as previously described (Tokay and Kockar 2016b). Protein concentrations were determined using the Bradford assay, adjusted to 4 µg/µL, and stored at −80 °C until use.

Biotin-labeled DNA probes containing HIF-1α binding sequences were prepared using the Pierce™ Biotin 3′ End DNA Labeling Kit (Thermo Fisher Scientific) according to the manufacturer’s instructions. Briefly, oligonucleotides (1 µM) were labeled using terminal deoxynucleotidyl transferase (TdT) in the presence of Biotin-11-UTP and incubated at 37 °C for 30 min. The reaction was terminated by addition of EDTA, and labeled probes were purified and stored at −20 °C. Prior to use, probes were denatured at 95 °C for 5 min. Binding reactions were performed in a total volume of 20 µL containing nuclear extract (5–10 µg), biotin-labeled probe, binding buffer, and poly(dI-dC) as a nonspecific competitor. For competition assays, a 100-fold molar excess of unlabeled (cold) probe was included to assess binding specificity. DNA-protein complexes were resolved on native polyacrylamide gels and transferred onto nylon membranes. Following UV crosslinking, detection was carried out using the Pierce™ LightShift Chemiluminescent EMSA Kit (Thermo Fisher Scientific) according to the manufacturer’s protocol. Signals were visualized using a chemiluminescence imaging system (Phusion FX, Vilber Lourmat) (Tokay and Kockar 2016b).

### Transient transfections and reporter assays

Cells were transiently transfected using the calcium phosphate precipitation method as previously described (Tokay and Kockar 2016a). For reporter assays, cells were seeded in 96-well plates and transfected with promoter-reporter constructs containing 5′-deletion fragments of the MIR100HG gene. Forty-eight to seventy-two hours post-transfection, conditioned medium was collected for analysis. Luciferase activity was measured using the Ready-To-Glow™ Secreted Luciferase Reporter Assay (Clontech/Takara Bio, USA), according to the manufacturer’s instructions. Briefly, 50 µL of conditioned medium was transferred to a 96-well plate, and 5 µL of 1× substrate/reaction buffer was added. Luminescence was measured using a microplate luminometer. Promoter activity was normalized to transfection efficiency using a co-transfected secreted alkaline phosphatase (SEAP) reporter system, and results were expressed as luciferase/SEAP ratios. Data were analyzed using GraphPad Prism software 9.0.

## Chromatin immunoprecipitation (ChIP) analysis

To evaluate DNA–protein interactions within the MIR100HG promoter region under hypoxic conditions, chromatin immunoprecipitation (ChIP) analysis was performed in Hep3B cells as previously described (Alper et al., 2025). Cells were cultured in 100-mm dishes until ~ 80% confluency and treated with 300 µM cobalt chloride (CoCl₂; Sigma-Aldrich, Cat#C8661) for 6 h to induce hypoxia. Cells were crosslinked with 1% formaldehyde (prepared from 37% stock solution) for 10 min at room temperature, and the reaction was quenched by the addition of 125 mM glycine. Following crosslinking, cells were washed with cold phosphate-buffered saline (PBS) containing protease inhibitor cocktail (Sigma-Aldrich, Cat#P8340), collected by scraping, and lysed in SDS lysis buffer. Chromatin was fragmented by sonication to yield DNA fragments in the range of 200–1000 bp. The lysates were centrifuged at 12,000 × g for 10 min at 4 °C, and the supernatant containing sheared chromatin was collected. For each immunoprecipitation reaction, chromatin equivalent to 1 × 10⁶ cells was used. ChIP assays were performed using the EZ-ChIP™ Chromatin Immunoprecipitation Kit (Millipore, Cat#17–371) according to the manufacturer’s instructions. Chromatin samples were diluted in ChIP dilution buffer and precleared with Protein G agarose beads to reduce nonspecific binding. Immunoprecipitation was carried out overnight at 4 °C with HIF-1α antibody. Immune complexes were captured using Protein G agarose beads, followed by sequential washing steps. A 10% input sample was reserved prior to immunoprecipitation for normalization. After reversal of crosslinks and DNA purification, enriched DNA fragments were analyzed by quantitative PCR (ChIP-qPCR). ChIP-qPCR data were quantified using the percent input (% input) method and normalized against IgG controls to assess specific transcription factor binding.

### Wound healing assay

Cell migration was evaluated using a wound healing (scratch) assay. Hepatocellular carcinoma cells were seeded in 6-well plates at a density of 5 × 10⁵ cells per well and allowed to reach approximately 90–100% confluency. A linear scratch was created across the cell monolayer using a sterile 200 µL pipette tip. Detached cells were removed by washing with phosphate-buffered saline (PBS), and cells were subsequently cultured under control or hypoxic conditions in serum-reduced medium to minimize proliferation effects. Images of the wound area were captured at 0, 3, 6, 24, and 48 h using an inverted phase-contrast microscope. Wound closure was quantified using ImageJ software (NIH, USA; RRID: SCR_003070) by measuring the wound area at each time point. Cell migration was expressed as the percentage of wound closure relative to the initial wound area at 0 h. All experiments were performed in triplicate (Hacıoğlu et al., 2020).

### Colony formation assay

The long-term proliferative capacity of cells was evaluated using a colony formation assay. Hep3B, SNU-398, and Clone-9 cells were seeded in 6-well plates at a density of 2 × 10³ cells per well and cultured under control or hypoxic conditions. After 10–14 days of incubation, cells were gently washed with phosphate-buffered saline (PBS) and fixed with 4% paraformaldehyde for 15 min at room temperature. Colonies were then stained with 0.5% methylene blue solution for 20 min and rinsed with distilled water to remove excess dye. Plates were air-dried and imaged using an inverted phase-contrast microscope (20× magnification). Colony numbers were compared with those of the control cells and hypoxia-treated wells (Hacıoğlu et al., 2020).

### TCGA-LIHC transcriptomic and survival analysis

Publicly available transcriptomic and clinical data from the TCGA Liver Hepatocellular Carcinoma (TCGA-LIHC) cohort were used to evaluate the expression profile and clinical relevance of MIR100HG. Normalized RNA-seq expression data (log2-transformed counts) and corresponding clinical annotations were obtained from the Genomic Data Commons (GDC) Data Portal. The samples were stratified into tumor and normal tissue groups for differential expression analysis. Comparisons between tumor and normal samples were performed using the Wilcoxon rank-sum test, and statistical significance was defined as *p* < 0.05. For survival analysis, patients were dichotomized into high and low MIR100HG expression groups based on the median expression value. Kaplan–Meier survival curves were generated, and differences in overall survival between the groups were evaluated using the log-rank test (Kaplan and Meier 1958). To investigate the relationship between MIR100HG expression and tumor progression, expression levels were further analyzed across pathological stages (Stages I–IV) using one-way ANOVA, followed by post hoc comparisons, where applicable. Correlation analyses between MIR100HG expression and hypoxia-related parameters, including hypoxia scores and HIF1A expression levels, were conducted using Spearman’s rank correlation coefficient (ρ) to account for the non-parametric data distribution (Spearman 1904). For transcriptomic profiling, differentially expressed genes (DEGs) associated with MIR100HG expression were identified by stratifying the samples into high- and low-expression groups. Genes with |log2 fold change| ≥ 1 and adjusted *p* < 0.05 were considered to be significant. Functional enrichment analyses were subsequently performed using Gene Ontology (GO) and pathway databases, including KEGG, and statistical significance was assessed using the Benjamini–Hochberg false discovery rate (FDR) correction (Benjaminit and Hochberg 1995). All statistical analyses and visualizations were performed using R software (version 4.5.3) with relevant packages, including ggplot2, survival, survminer, and clusterProfiler (R: The R Project for Statistical Computing n.d.; Yu et al. 2012). Data are presented as median ± interquartile range or mean ± standard deviation, as appropriate.

### RNA seq analysis

Hep3B cells were cultured under normoxic and hypoxic conditions, and total RNA was isolated from each condition. In addition to comparing normoxia and hypoxia, transcriptomic profiling was also performed under conditions where MIR100HG was silenced to evaluate MIR100HG-dependent transcriptional regulation. Specifically, Hep3B cells were subjected to silencing of the MIR100HG gene under hypoxic conditions (hypoxia + sh-MIR100HG) and compared with the unsilencing hypoxia group. Libraries prepared from the obtained RNAs were sequenced on the Illumina platform, and the raw data were evaluated using FastQC (RRID: SCR_014583) for quality control. Adapter cleaning and removal of low-quality bases were performed using the Trimmomatic (RRID: SCR_011848) tool (Bolger et al. 2014). The cleaned sequences were mapped to the human reference genome (GRCh38) using STAR aligner (RRID: SCR_004463) (Dobin et al. 2013), and gene expression counts were determined using featureCounts (Liao et al. 2014). Differential gene expression analysis was performed using the DESeq2 (RRID: SCR_015687) package (Love et al. 2014), and heat maps and volcano plots were generated from the obtained data after variance stabilization transformation (VST). For significant differential expression, threshold values of p-adj < 0.05 and |log₂FC| ≥ 1 were used. Functional enrichment analyses were performed using the clusterProfiler (RRID: SCR_016884) (Yu et al. 2012) and ReactomePA (RRID: SCR_019316) (Moore and Schmitz 2016) packages; KEGG (RRID: SCR_012773), Gene Ontology (RRID: SCR_002811) (Biological process, Cellular component, and Molecular function), and Reactome pathway analyses were performed, and the results were visualized with bar plot and dot plot graphics.

### MIR100HG Overexpression and Knockdown studies

For gain-of-function experiments, the pcDNA3.1-LincNed125 (MIR100HG) overexpression plasmid (MIR100HG-OE) has been obtained from Dr. Elisa Caffarelli (Institute of Molecular Biology and Pathology, CNR, Rome, Italy) (Bevilacqua et al. 2015). Transfection was performed using Lipofectamine 3000 (Thermo Fisher Scientific) according to the manufacturer’s protocol. Briefly, the cells were seeded at 60–70% confluence and incubated with plasmid–lipid complexes for 24–48 h. Successful overexpression was confirmed by qRT-PCR using MIR100HG-specific primer.

Loss-of-function studies were conducted using shRNA constructs targeting MIR100HG (sh-MIR100HG) cloned into lentiviral pLKO.1. A scrambled shRNA sequence (SCR) was used as a negative control. Lentiviral particles were produced in HEK293T cells and used to infect target cells in the presence of 8 µg/mL protamine sulfate. After 48 h, the transduced cells were selected using 0.5 µg/mL puromycin for 72 h. The knockdown efficiency was validated using qRT-PCR.

### Statistical analyses

All experiments were performed with at least three independent, biological replicates. Quantitative data are presented as mean ± standard deviation (SD). In the analysis of differences between groups, a two-tailed Student’s t-test was used for two-group comparisons, and one-way analysis of variance (ANOVA) was used for three or more group comparisons. All statistical analyses were performed using GraphPad Prism (v10.2) software. Statistical significance was set at *p* < 0.05.

## Results

### MIR100HG is clinically associated with hypoxia and disease progression in HCC

**Fig. 1:**
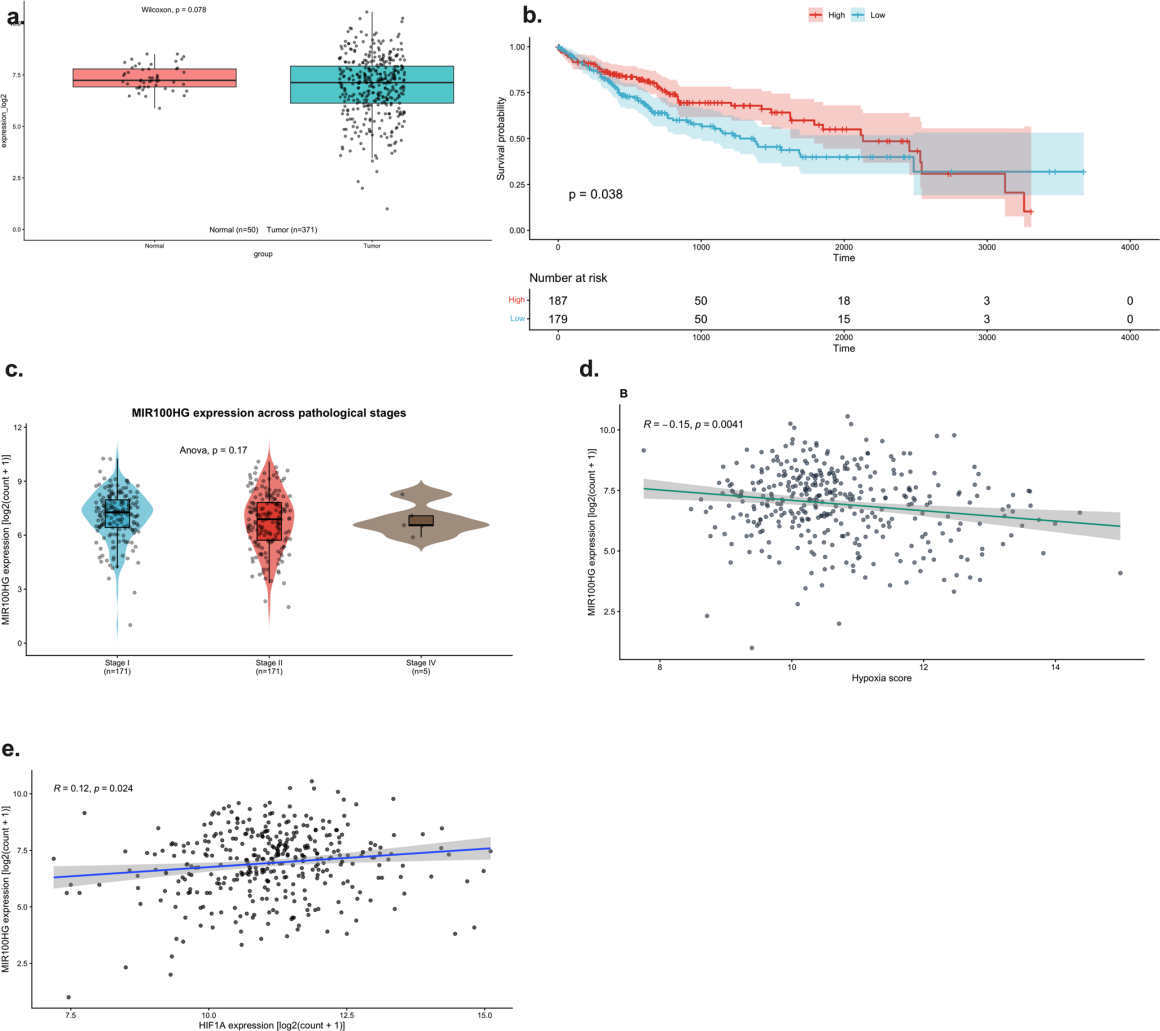
MIR100HG is associated with tumor progression and hypoxia in hepatocellular carcinoma (TCGA-LIHC). (**a**) Differential expression of MIR100HG between normal liver tissues (n = 50) and HCC tumor samples (n = 371). Statistical significance was assessed using the Wilcoxon rank-sum test. (**b**) Kaplan–Meier overall survival analysis of HCC patients stratified by MIR100HG expression (high vs. low). Survival differences were evaluated using the log-rank test. (**c**) MIR100HG expression across pathological stages (Stages I–IV). Statistical analyses were performed using one-way ANOVA. The sample size for stage IV was limited (n = 5). (**d**) Correlation between MIR100HG expression and hypoxia score in TCGA-LIHC samples, assessed using Spearman’s correlation analysis. (**e**) Correlation between MIR100HG and HIF1A expression levels, evaluated using Spearman’s correlation analysis

To investigate the clinical relevance of MIR100HG in hepatocellular carcinoma, we first analyzed its expression in the TCGA-LIHC cohort. MIR100HG expression was significantly different between tumor and normal tissues (Fig. 1a). Survival analysis revealed that patients with higher MIR100HG expression exhibited significantly poorer overall survival than those with lower expression levels (Fig. 1b). Next, we examined MIR100HG expression across different pathological stages. Although a trend toward differential expression was observed, no statistically significant differences were detected among the stages, potentially due to the limited sample size of advanced-stage patients (Fig. 1c). Given the known role of hypoxia in HCC progression, we assessed the relationship between MIR100HG expression and hypoxia-associated features. MIR100HG expression showed a significant correlation with hypoxia scores (Fig. 1d), as well as with HIF1A expression levels (Fig. 1e), suggesting a potential link between MIR100HG and hypoxia-driven transcriptional programs in HCC.

### Validation of hypoxic conditions in diverse cell models

**Fig. 2:**
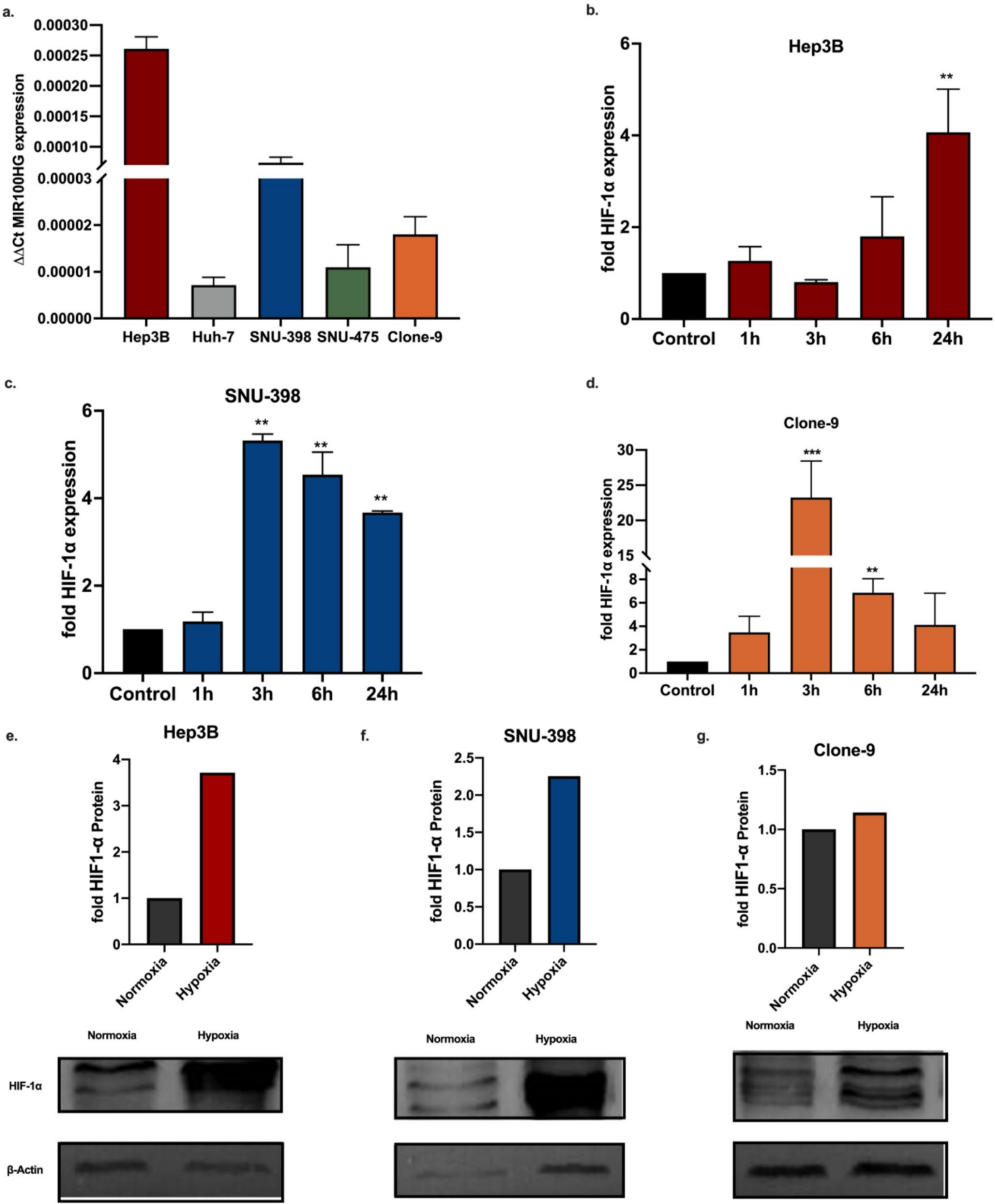
Hypoxia induces HIF-1α stabilization and MIR100HG expression across hepatocellular carcinoma and non-tumor hepatocyte models. (**a**) Basal expression levels of MIR100HG in hepatocellular carcinoma cell lines (Hep3B, Huh-7, SNU-398, and SNU-475) and non-tumor hepatocyte-derived Clone-9 cells, as determined by qRT-PCR. (**b**, **c** and **d**) Time-course analysis of HIF-1α mRNA expression under hypoxic conditions in Hep3B (primary model), SNU-398 (validation model), and Clone-9 (non-tumor control) cells. Cells were exposed to hypoxia for the indicated time points (1, 3, 6, and 24 h), and relative HIF-1α expression levels were quantified. A time-dependent induction of HIF-1α expression was observed, with distinct response dynamics across malignant and non-malignant models. (**e**, **f** and **g**) Quantification of HIF-1α protein levels in normoxic and hypoxic Hep3B, SNU-398, and Clone-9 cells, as determined by densitometric analysis of immunoblotting data. Representative immunoblots showing HIF-1α protein accumulation under hypoxia, with β-actin as a loading control. Hep3B cells were used as a hepatocellular carcinoma model, SNU-398 cells as an independent validation model, and Clone-9 cells as a non-tumor hepatocyte-derived control. Data are presented as mean ± SD from independent experiments. Statistical significance was determined using appropriate tests (*p < 0.05, **p < 0.01, ***p < 0.001)

A stepwise experimental strategy was employed, beginning with the broad screening of multiple hepatic cell lines, followed by focused validation in selected models, and ultimately progressing to detailed mechanistic analyses in the most responsive systems. To capture the biological heterogeneity of hepatocellular carcinoma (HCC), four liver cancer-derived cell lines (Hep3B, Huh-7, SNU-398, and SNU-475) and a non-tumorigenic hepatocyte-derived cell line (Clone-9) were used. Hep3B and Huh-7 cells represent epithelial-like HCC phenotypes, whereas SNU-398 and SNU-475 cells exhibit mesenchymal-like characteristics, and Clone-9 cells serve as a physiological reference model. To establish baseline expression patterns, we quantified MIR100HG mRNA levels under normoxic conditions (Fig. 2a). Among all the tested models, Hep3B cells exhibited markedly elevated MIR100HG expression compared to Huh-7, SNU-398, SNU-475, and Clone-9 cells (*p* < 0.001), indicating a pronounced tumor-associated expression profile. In contrast, mesenchymal-like cell lines displayed moderate expression levels, whereas Clone-9 cells showed minimal expression. Notably, the expression patterns observed in Huh-7 and SNU-475 cells are presented in Supplementary Fig. 1, providing additional support for the heterogeneity of MIR100HG expression across the HCC subtypes. To validate the establishment of hypoxic conditions, we assessed HIF-1α mRNA expression in a time-dependent manner following CoCl₂ treatment. In Hep3B cells (Fig. 2b), HIF-1α expression increased progressively, reaching a statistically significant peak at 24 h (*p* < 0.01 vs. control), consistent with the sustained hypoxic adaptation. In contrast, SNU-398 cells (Fig. 2c) exhibited a rapid and transient response, with maximal induction observed at 3 h (*p* < 0.01), followed by a gradual decline in expression at later time points. Importantly, similar hypoxia-induced HIF-1α dynamics were also observed in Huh-7 and SNU-475 cells (Supplementary Fig. 1), further confirming the robustness of hypoxia induction across distinct cellular contexts.

Collectively, these findings validate the successful establishment of hypoxic conditions across all tested hepatic models and reveal cell type–specific differences in both basal MIR100HG expression and hypoxia responsiveness. Based on these results, Hep3B cells were selected as the primary experimental model because of their high basal expression and sustained hypoxic response, whereas SNU-398 cells were employed as a complementary system to represent mesenchymal-like dynamics. Clone-9 cells were retained as non-malignant references for subsequent functional and mechanistic analyses.

### Functional characterization of hypoxia effects in hepatocellular cell models

**Fig. 3:**
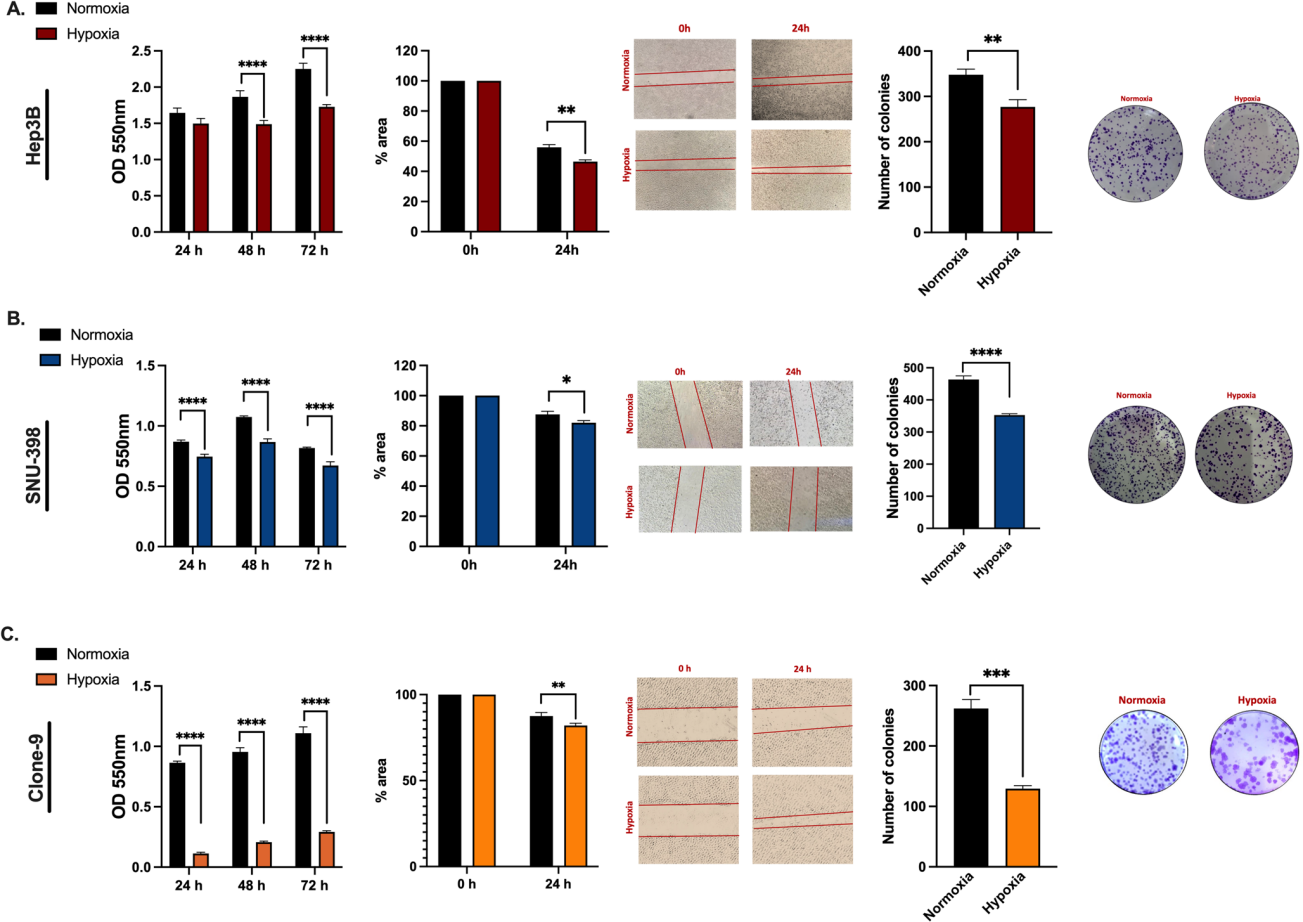
Functional analysis of hypoxic effects in hepatic cell models with distinct phenotypes. Epithelial-like hepatocellular carcinoma cells (Hep3B) (**A**), mesenchymal-like hepatocellular carcinoma cells (SNU-398) (**B**), and healthy rat hepatocyte cells (Clone 9) (**C**) were cultured under normoxic and hypoxic conditions. Cell proliferation was assessed using the MTT assay at 24, 48, and 72 h (left panels). Migration ability was evaluated using a wound healing assay at 0 h and 24 h, with quantification of the wound closure percentage and representative images (middle panels). Clonogenic potential was determined using a colony formation assay, with quantification of colony numbers and representative crystal violet-stained images (right panels). Data are presented as mean ± SD from a minimum of three independent experiments. Statistical significance was determined using two-way ANOVA for proliferation data and unpaired t-test for migration and colony assays (*p < 0.05, **p < 0.01, ***p < 0.001, ****p < 0.0001)

To determine how hypoxic stress modulates key tumor-related cellular functions, proliferation, migration, and clonogenic capacity were systematically evaluated across epithelial-like (Hep3B), mesenchymal-like (SNU-398), and non-tumorigenic (Clone-9) hepatic cell models under normoxic and hypoxic conditions. In epithelial-like Hep3B cells (Fig. 3a), hypoxia resulted in marked suppression of proliferative capacity, with significant reductions observed at 48 and 72 h (*p* < 0.0001). This anti-proliferative effect was accompanied by a pronounced decrease in migratory activity, as demonstrated by impaired wound closure at 24 h (*p* < 0.01) and a significant reduction in clonogenic potential (*p* < 0.01). These findings indicate that, despite their tumorigenic nature, epithelial-like HCC cells exhibit limited functional adaptability to sustained hypoxic stress. In contrast, mesenchymal-like SNU-398 cells (Fig. 3b) displayed a partially attenuated response. Although proliferation was significantly reduced (*p* < 0.0001), the inhibitory effect on migration was comparatively moderate (*p* < 0.05), suggesting a retained capacity for motility under hypoxic conditions. Clonogenic survival was also significantly impaired (*p* < 0.0001), supporting the notion that mesenchymal-like phenotypes may exhibit differential sensitivity to hypoxia, particularly in processes related to cellular plasticity and survival. Notably, the most pronounced effects were observed in the non-tumorigenic Clone-9 cells (Fig. 3c), where hypoxia induced a substantial reduction in proliferation (*p* < 0.0001), migration (*p* < 0.01), and colony formation (*p* < 0.001) compared to normoxia. This heightened sensitivity suggests that normal hepatocytes lack the adaptive mechanisms required to tolerate hypoxic stress, in contrast to cancer cells, which may partially preserve their functional capacity under adverse microenvironmental conditions. Collectively, these results revealed a hierarchical pattern of hypoxia sensitivity across hepatic cell models, with non-malignant cells being the most vulnerable, followed by mesenchymal-like and epithelial-like cancer cells. This differential response underscores the context-dependent nature of hypoxic adaptation and suggests that tumor cells retain selective advantages that enable partial functional resilience under hypoxic conditions.

### *In silico* mapping of the MIR100HG promoter architecture and variant profiling

**Fig. 4:**
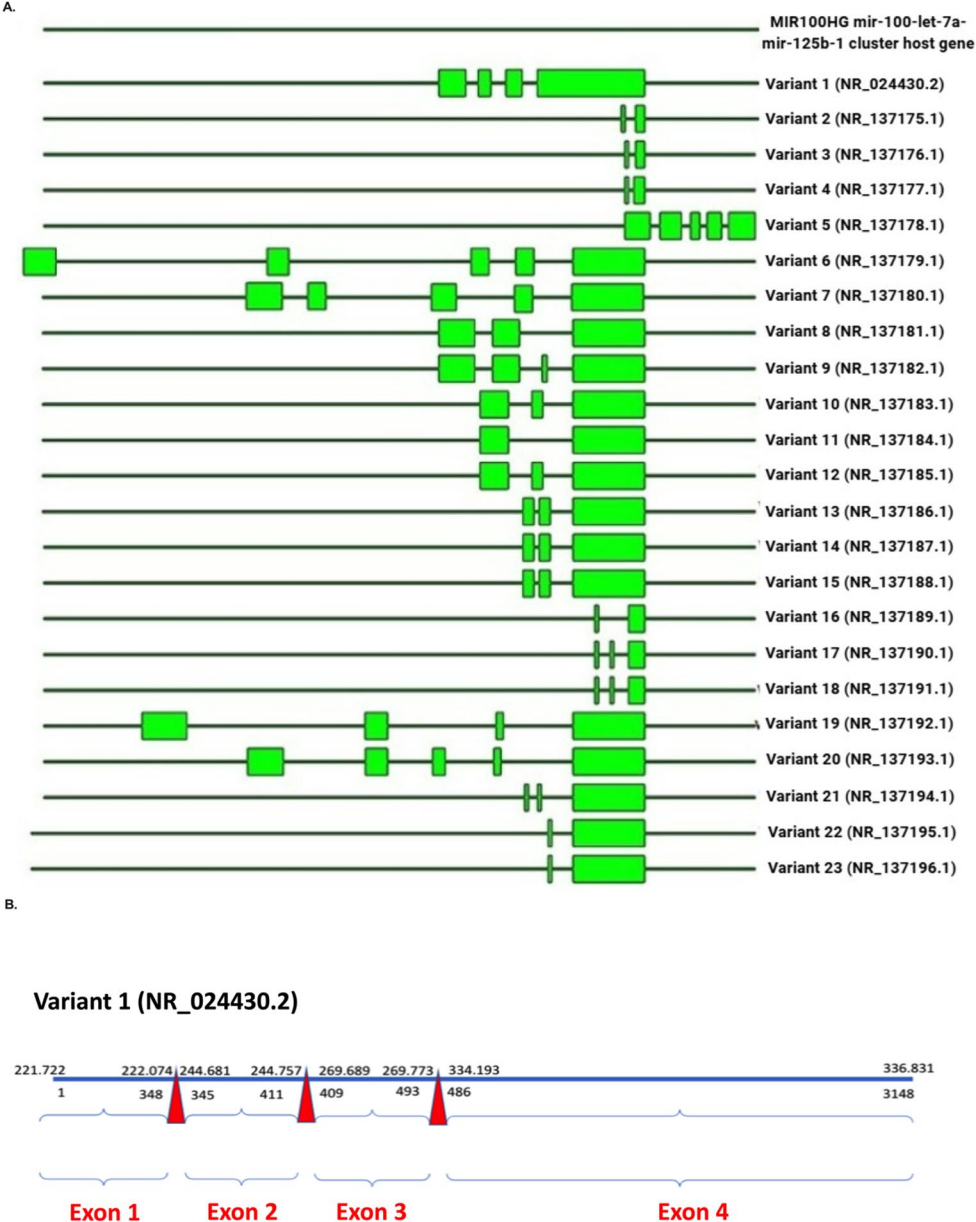
*In silico* **MIR100HG Gene Analysis** Bioinformatic analysis of MIR100HG was performed. **(A)** A combined representation of 23 different variants of the MIR100HG gene was used to detail the variants. Data were obtained from the NCBI. **(B)** An illustration of variant 1, cloned in this study, is shown

A comprehensive in silico analysis was performed to characterize the genomic organization and promoter architecture of the MIR100HG gene. MIR100HG is located on chromosome 11q24.1 (122,028,329–122,422,871) and exhibits extensive transcript diversity, with 23 annotated transcript variants identified in the NCBI database (Fig. 4A). These variants display heterogeneity in their 5′ transcription start sites, suggesting alternative promoter usage and complex, transcriptional regulation. The exonic regions for each variant are indicated in green and mapped relative to the full genomic locus. Transcript variant 1 (NR_024430.2) was selected as the reference transcript for subsequent promoter analyses because of its annotation status and structural completeness. This transcript spanned 3148 bp and consisted of four exons (Fig. 4B). Variants 1, 8, and 9 originated from overlapping genomic regions, supporting the existence of a shared core promoter. Based on the genomic structure of variant 1, a putative promoter region encompassing 1013 bp upstream and 231 bp downstream of the transcription start site (+ 1) was defined, yielding a total length of 1244 bp. This region was selected for downstream functional and structural characterizations. To investigate the sequence composition of the predicted promoter, GC content analysis was performed using EMBOSS CpGPlot software. The overall GC content of the 1244 bp region was approximately 50%, with no classical CpG islands. However, a localized increase in GC content (> 60%) was observed between positions − 800 and − 1000, suggesting the presence of GC-rich regulatory elements (Supplementary Fig. 2). Further in silico analysis of transcription factor binding sites revealed multiple potential regulatory motifs within the promoter sequence, including binding sites for SP1, Krüppel-like factors (KLF), CCAAT/enhancer-binding proteins (C/EBP), upstream stimulatory factor (USF), and STAT family transcription factors. The presence of these elements indicates that MIR100HG transcription may be regulated through diverse signaling pathways, supporting a multifactorial regulatory mechanism. Collectively, these findings provide a structural and regulatory framework for MIR100HG transcriptional control and establish a basis for the subsequent experimental validation of promoter activity.

### HIF-1α directly binds to and activates the MIR100HG promoter under hypoxic conditions

**Fig. 5:**
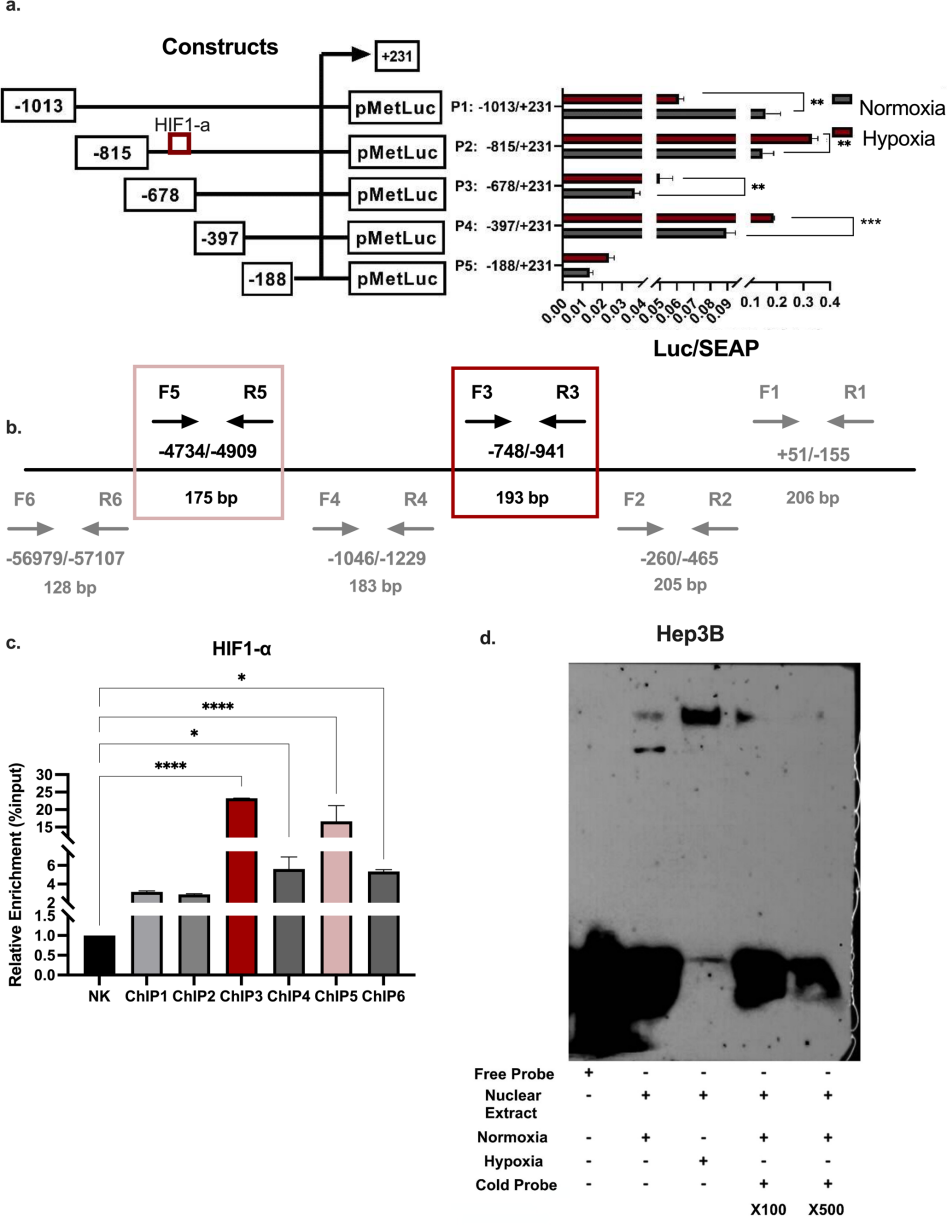
HIF-1α directly binds to and transcriptionally activates the MIR100HG promoter in hypoxic conditions. (**A**) Schematic representation of MIR100HG promoter deletion constructs cloned into luciferase reporter vectors. The putative HIF-1α binding site (hypoxia response element, HRE) is indicated. (**B**) Luciferase reporter assay showing the transcriptional activity of MIR100HG promoter constructs under normoxic and hypoxic conditions. Deletion of the HRE-containing region significantly reduced hypoxia-induced promoter activity, indicating the functional relevance of the HIF-1α binding site. (**C**) Schematic overview of the primer sets spanning the MIR100HG promoter region used for chromatin immunoprecipitation (ChIP) analysis. The region corresponding to the predicted HIF-1α binding site is highlighted. (**D**) Electrophoretic mobility shift assay (EMSA) demonstrating the direct binding of nuclear proteins to the MIR100HG promoter probe. A shifted band corresponding to the HIF-1α–DNA complex was observed under hypoxic conditions, and its specificity was confirmed by competition with an excess unlabeled (cold) probe. (**E**) ChIP-qPCR analysis of HIF-1α occupancy at the MIR100HG promoter. Significant enrichment was observed in the region encompassing the predicted HRE compared to negative control regions (NK), confirming the in vivo binding of HIF-1α to the MIR100HG promoter. Data are presented as mean ± SD from independent experiments. Statistical significance was determined using appropriate tests (**p < 0.01, ***p < 0.001, ****p < 0.0001)

To identify hypoxia-responsive regulatory regions within the MIR100HG promoter and determine the contribution of HIF-1α, an integrated approach combining luciferase reporter assays, chromatin immunoprecipitation (ChIP–qPCR), and electrophoretic mobility shift assay (EMSA) was employed in Hep3B cells exposed to CoCl₂-induced hypoxia. Promoter deletion analysis revealed that constructs containing the distal promoter region (P1: −1013/+231 and P2: −815/+231) exhibited significant induction under hypoxic conditions compared to normoxic conditions (*p* < 0.01), indicating the presence of functional hypoxia-responsive elements (HREs) within this region (Fig. 5A). Notably, shorter promoter fragments lacking canonical HRE motifs (P3–P5) also retained hypoxic responsiveness, albeit at reduced levels (P3, *p* < 0.01; P4–P5, *p* < 0.001). These findings suggest that MIR100HG transcription is regulated not only through classical HIF-1α–dependent mechanisms but also via additional hypoxia-sensitive regulatory elements located within the proximal promoter region. To directly assess HIF-1α occupancy, ChIP–qPCR analysis was performed across six regions spanning the promoter (ChIP1–ChIP6). Among these, the − 748/−941 region (ChIP3) displayed the strongest enrichment (~ 22% input), significantly exceeding both the negative control and all other regions (****p < 0.0001) (Fig. 5C). Moderate secondary enrichment was also detected at the distal − 4734/−4909 region (ChIP5), indicating the presence of an additional upstream interaction site. Importantly, the region exhibiting maximal HIF-1α binding (− 748/−941) corresponded precisely to the core hypoxia-responsive segment identified in luciferase assays, demonstrating a strong concordance between functional promoter activity and transcription factor occupancy. This overlap defines the − 748/−941 interval as the principal regulatory hotspot that mediates hypoxia-induced MIR100HG activation. To further validate the direct DNA–protein interaction, EMSA was performed using nuclear extracts from normoxic and hypoxic Hep3B cells (Fig. 5D). A distinct DNA–protein complex was observed under hypoxic conditions, which was markedly reduced upon competition with excess unlabeled (cold) probe in a dose-dependent manner (100× and 500×), confirming the binding specificity. Increased complex formation under hypoxia further supports the enhanced nuclear accumulation and DNA-binding activity of HIF-1α. Collectively, these results demonstrate that HIF-1α directly binds to the MIR100HG promoter and transcriptionally activates its expression in hypoxic conditions. While the − 748/−941 region constitutes the primary HIF-1α interaction site, additional distal and proximal elements contribute to a multilayered regulatory architecture, indicating that MIR100HG transcription is governed by both canonical HIF-dependent and auxiliary hypoxia-responsive mechanisms.

### Transcriptomic profiling of MIR100HG-associated gene expression changes in Hep3B cells under normoxic and hypoxic conditions

**Fig. 6:**
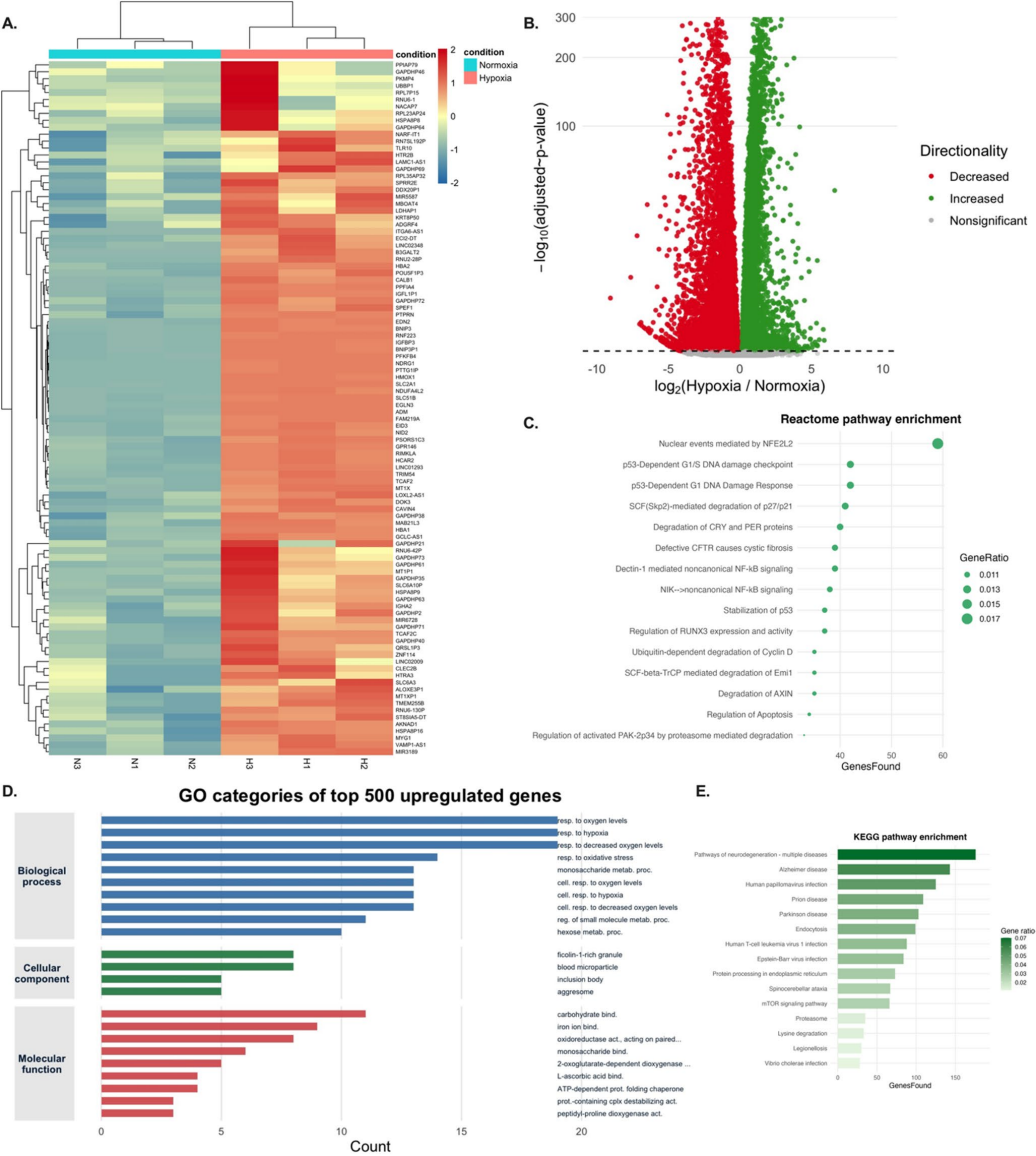
Transcriptomic analysis of MIR100HG expression in Hep3B cells under normoxic and hypoxic conditions. (**A**) Heatmap of differentially expressed genes (DEGs) showing the significant upregulation of MIR100HG under hypoxia. (**B**) Volcano plot of DEGs from the DESeq2 analysis (red = downregulated, green = upregulated, gray = not significant). (**C**) Reactome enrichment analysis highlighting pathways related to the hypoxia response, NF-κB signaling, and cell cycle. (**D**) KEGG enrichment analysis showing the pathways linked to stress, infection, and metabolism. (**E**) GO enrichment of upregulated genes indicated oxygen response and hypoxia-related processes, consistent with increased MIR100HG expression levels

To characterize the global transcriptional response associated with MIR100HG under hypoxic conditions, RNA-sequencing-based differential expression analysis was performed comparing normoxic and CoCl₂-induced hypoxic Hep3B cells. Unsupervised hierarchical clustering revealed a clear separation between normoxic and hypoxic samples, indicating robust hypoxia-driven transcriptional reprogramming (Fig. 6A). Hypoxia induced a distinct gene expression signature characterized by widespread upregulation of oxygen-responsive and stress-associated genes, whereas normoxic samples exhibited a comparatively stable transcriptional profile. Volcano plot analysis further demonstrated a substantial number of significantly deregulated genes under hypoxia, with a dominant shift toward upregulation of genes (Fig. 6B). These transcriptional changes were statistically robust, as indicated by high −log10(adjusted p-value) distributions, and primarily involved genes associated with metabolic adaptation, oxygen homeostasis, and stress responses. Pathway enrichment analysis using Reactome revealed that hypoxia activates multiple interconnected regulatory networks (Fig. 6C), including the DNA damage response, p53 signaling, NF-κB–mediated inflammatory pathways, and ubiquitin-dependent protein degradation. These pathways are consistent with the adaptive mechanisms that promote cellular survival under low-oxygen conditions. Gene Ontology (GO) enrichment analysis of the top upregulated genes highlighted significant overrepresentation of biological processes related to hypoxia response, reactive oxygen species regulation, metabolic adaptation, and protein homeostasis (Fig. 6D). At the cellular component level, enrichment was observed in organelles associated with stress adaptation, including the endoplasmic reticulum, mitochondria, and cytoplasmic protein complexes. The molecular function categories emphasized oxidoreductase activity, protein binding, and enzymatic regulation. Consistently, KEGG pathway analysis identified significant enrichment in HIF-1 signaling, p53 signaling, apoptosis, glycolysis, and NF-κB–associated pathways (Fig. 6E), indicating the coordinated activation of metabolic and survival programs under hypoxia. Collectively, these findings demonstrate that hypoxia induces extensive transcriptomic remodeling in Hep3B cells, with MIR100HG-associated gene networks centrally involved in the regulation of cellular adaptation, stress tolerance, and metabolic reprogramming.

### GSEA-based identification of hypoxia-driven MIR100HG-associated pathways in Hep3B cells

**Fig. 7:**
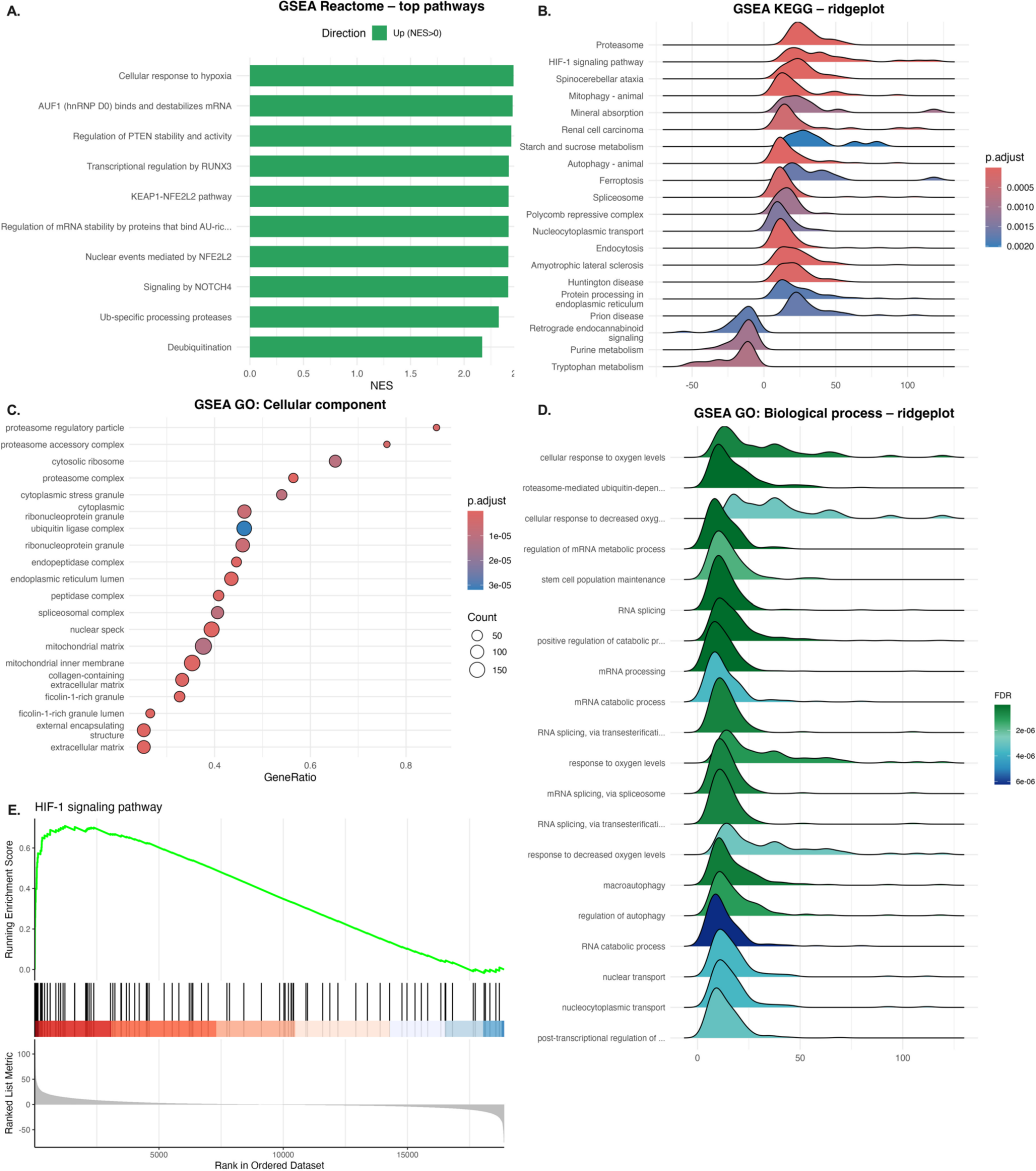
GSEA of MIR100HG-associated genes in Hep3B cells under hypoxia. (**A**) Reactome, (**B**) KEGG, and (**C**) GO biological process ridge plots; **(D**) enrichment curve plots for sample pathways; (**E**) GO cellular component dot plot. Under hypoxia, HIF-1 signaling, KEAP1–NFE2L2, proteasome, and oxygen response-related pathways were significantly enriched

To further elucidate the functional landscape of MIR100HG-associated transcriptional changes, gene set enrichment analysis (GSEA) was performed using the Reactome, KEGG, and Gene Ontology (GO) databases. Reactome-based GSEA revealed strong enrichment of hypoxia-responsive pathways, with “cellular response to hypoxia” showing the highest normalized enrichment score (NES), confirming the robustness of the hypoxic transcriptional program (Fig. 7A). In parallel, pathways related to post-transcriptional regulation, including AU-rich element–mediated mRNA stability and RNA-binding protein activity, were significantly enriched, indicating extensive regulation at the RNA level. GSEA–KEGG analysis demonstrated prominent enrichment of the proteasome and HIF-1 signaling pathways, highlighting the activation of protein quality control and canonical hypoxia signaling (Fig. 7B). Additionally, pathways associated with autophagy, mitophagy, and ferroptosis were significantly enriched, suggesting the activation of stress adaptation and regulated cell death mechanisms. Metabolic pathways, including purine and tryptophan metabolism, were upregulated, reflecting metabolic reprogramming under hypoxia. GO cellular component analysis revealed significant enrichment of protein degradation and RNA-processing structures, including proteasome complexes, ubiquitin ligase complexes, and cytoplasmic stress granules (Fig. 7C). Furthermore, the enrichment of the endoplasmic reticulum, mitochondrial matrix, and ribonucleoprotein complexes indicates the coordinated regulation of protein folding, transport, and metabolic adaptation. GO biological process analysis confirmed strong enrichment in hypoxia-related processes, including the cellular response to oxygen levels, response to decreased oxygen levels, and regulation of hypoxia-responsive pathways (Fig. 7D–E). In addition, pathways related to RNA processing, splicing, mRNA turnover, and nucleocytoplasmic transport were significantly activated, suggesting that post-transcriptional regulation is a major component of the hypoxic response. Taken together, the GSEA results demonstrated that hypoxia drives the coordinated activation of transcriptional and post-transcriptional regulatory networks, with MIR100HG-associated gene signatures strongly linked to HIF-1 signaling, oxidative stress response, protein homeostasis, and metabolic adaptation pathways. Key hypoxia response processes, such as cellular response to oxygen levels, response to decreased oxygen levels, and positive regulation of cellular response to hypoxia, showed the highest enrichment. Additionally, post-transcriptional regulatory mechanisms, such as mRNA processing, RNA splicing, mRNA catabolic processes, and nucleocytoplasmic transport, were also significantly enriched. Increases were also observed in processes reflecting protein quality control and stress adaptation, such as the ribosome-mediated ubiquitin-dependent protein catabolic process and regulation of autophagy.

### Experimental validation of MIR100HG-driven hypoxia-associated transcriptional network

**Fig. 8:**
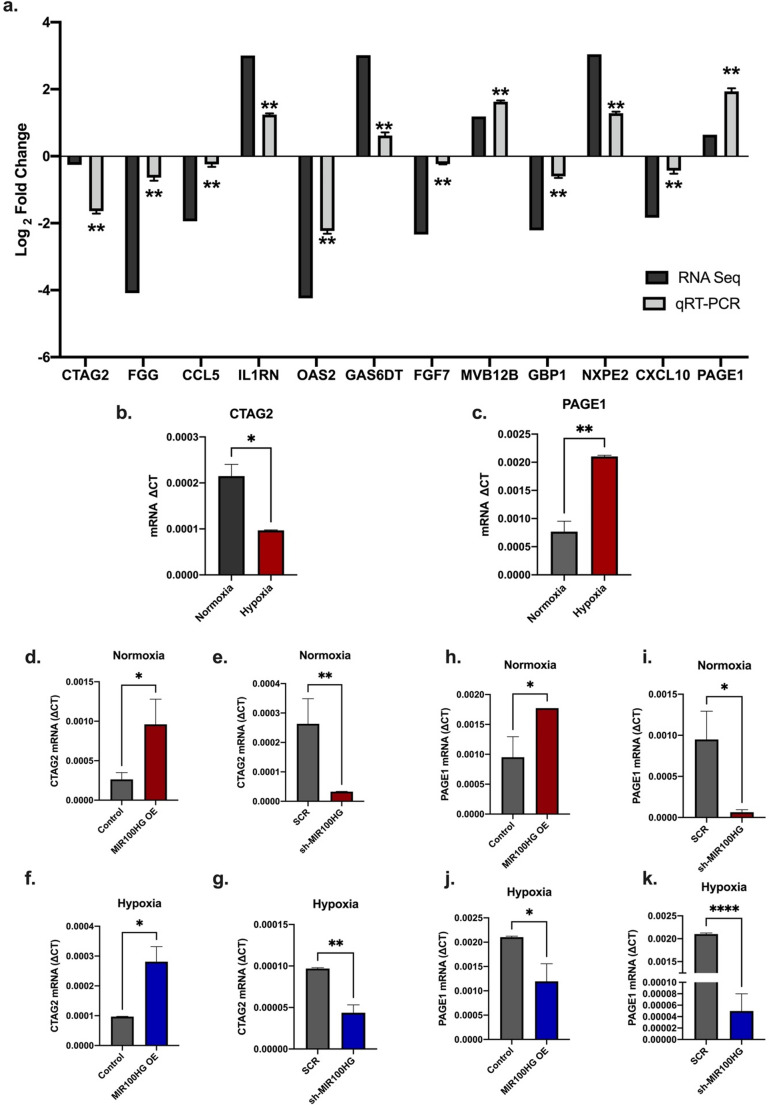
Validation of hypoxia-responsive gene expression and MIR100HG-dependent regulation of target genes. (**A**) RNA-seq and qRT-PCR comparison of log₂ fold-changes for genes differentially expressed under hypoxia in Hep3B cells, normalized to β2-microglobulin expression. (**B–>C**) Hypoxia significantly altered CTAG2 and PAGE1 mRNA levels. (**D, E, F **and **G**) MIR100HG overexpression enhanced CTAG2 expression under normoxic and hypoxic conditions, whereas MIR100HG knockdown markedly suppressed CTAG2 expression. (**H**, **I**, **J**, and **K**) PAGE1 exhibited an analogous MIR100HG-dependent regulatory pattern. Together, these findings validate the transcriptomic dataset and reveal a direct functional contribution of MIR100HG to the regulation of CTAG2 and PAGE1 transcription in different oxygen environments. Statistical significance: *p < 0.05, **p < 0.01, ***p < 0.0001

Building upon the global transcriptomic and pathway enrichment analyses (Figs. 6 and 7), which demonstrated that hypoxia induces a coordinated activation of adaptive gene networks, we next sought to experimentally validate key differentially expressed genes and functionally assess their dependence on MIR100HG. To this end, selected hypoxia-responsive genes identified by RNA-sequencing-including CTAG2, PAGE1, IL1RN, GAS6DT, CXCL10, GBP1, and OAS2, were subjected to qRT-PCR validation. Comparative analysis revealed a high degree of concordance between RNA-seq-derived log₂ fold-change values and qRT-PCR measurements (Fig. 8A), confirming the robustness and reproducibility of the hypoxia-induced transcriptional signature. Among these candidates, CTAG2 and PAGE1 were selected for further investigation because of their known association with cancer/testis antigens and their emerging roles in tumor immune modulation and hypoxia adaptation. Consistent with the transcriptomic data, both genes exhibited significant changes in expression under hypoxic conditions (Fig. 8B–C), supporting their classification as hypoxia-responsive targets. To determine whether these transcriptional changes were directly regulated by MIR100HG, gain- and loss-of-function experiments were performed under both normoxic and hypoxic conditions. Under normoxia, MIR100HG overexpression significantly increased CTAG2 and PAGE1 expression, whereas MIR100HG silencing resulted in a marked reduction in both transcripts (Fig. 8D-E, H-I). Notably, this regulatory effect was amplified under hypoxic conditions. Specifically, MIR100HG overexpression enhanced CTAG2 and PAGE1 expression under hypoxic conditions (Fig. 8F, J), whereas MIR100HG knockdown led to a pronounced suppression of these genes (Fig. 8G, K), indicating a hypoxia-dependent strengthening of MIR100HG-mediated transcriptional regulation. These findings demonstrate that MIR100HG functionally modulates the expression of key hypoxia-responsive genes. Importantly, while hypoxia induces broad transcriptomic reprogramming, only a subset of these genes appears to be sensitive to MIR100HG perturbation, suggesting that MIR100HG acts as a context-dependent regulatory component within the hypoxia-associated transcriptional network rather than a universal master regulator. In addition to cancer/testis antigens, several validated targets, including IL1RN, OAS2, CXCL10, and GBP1, are implicated in immune signaling and inflammatory responses, suggesting that MIR100HG may contrubite to the coordination of stress adaptation and tumor-immune interactions under hypoxic conditions. Taken together, the integration of RNA-seq, pathway enrichment, and targeted validation experiments supports a model in which MIR100HG contributes to hypoxia-associated transcriptional reprogramming by modulating specific components of adaptive gene networks, thereby influencing cellular plasticity in hepatocellular carcinoma under hypoxis stress.

## Discussion

In recent years, long non-coding RNAs (lncRNAs) have gained considerable importance owing to their roles in cancer biology, and among them, MIR100HG stands out as a polycistronic lncRNA frequently dysregulated in human cancers. Although previous studies have reported its oncogenic role and elevated expression in hepatocellular carcinoma (Li et al., 2021; Wu et al. 2022), the regulatory mechanisms governing MIR100HG under hypoxic conditions, a hallmark of the hepatocellular carcinoma (HCC) microenvironment, remain largely unexplored. Despite the fact that hypoxia is a major microenvironmental feature of HCC, understanding how MIR100HG is integrated into hypoxia-driven transcriptional programs represents a critical gap in the literature. Ma et al. (2018) identified four different lncRNAs, including MIR100HG, by analyzing the lncRNA expression of 364 HCC patients using the Cancer Genome Atlas dataset. MIR100HG expression studies were performed in four HCC cell lines (MHCC97L, SMCC-7721, Huh-7, and MHCC97H) and one healthy liver cell line (LO2), and it was determined that there was an increase in MIR100HG expression in all HCC cell models compared to healthy liver cells (Ma et al. 2018). In another study by Li et al. (2021), MIR100HG expression was studied in Hep3B, HepG2, SK-HEP1, and Huh-7 cell lines (Li et al., 2021). Since HCC commonly develops in fibrotic and cirrhotic tissue where vascularization is impaired, the resulting oxygen deprivation strongly activates HIF-1α-mediated transcriptional programs (Choi et al. 2017; Semenza 2010). Therefore, determining how MIR100HG responds to hypoxia constitutes a significant gap in the literature and is the core motivation for this study.

Our findings demonstrated that MIR100HG was expressed in all liver cell lines examined and was most abundant in Hep3B cells under normoxic conditions. Importantly, MIR100HG expression was consistently induced under hypoxic conditions across both malignant and non-malignant hepatic models, suggesting that MIR100HG is an integral component of the cellular response to hypoxia. This observation indicates that MIR100HG may function beyond tumor-specific regulation and instead participate in a broader hypoxia-adaptive transcriptional program. Consistent with our findings, MIR100HG has been identified as a hypoxia-induced differentially expressed lncRNA (Ou et al. 2026). According to the literature, MIR100HG has been shown to play an important role in various cellular processes and disease pathogenesis under hypoxic conditions. In the context of Pulmonary Arterial Hypertension (PAH), MIR100HG has been found to increase cellular proliferation and reduce apoptosis in response to CoCl2-induced hypoxia. Studies suggest that MIR100HG may play a critical role in PAH pathogenesis and may be a potential therapeutic target. Mechanistically, MIR100HG was found to have a strong association with hub genes such as LDHA, PFKP, and VEGFA, which are closely related to the cellular response to hypoxia and the regulation of glycolysis. Overall, these findings highlight that MIR100HG contributes to disease development through its regulatory effects on cellular energy metabolism and proliferation in hypoxic environments (Chen et al. 2020).

Gene regulatory networks in hepatocellular carcinoma are highly dynamic and evolve during disease progression. Transcriptional modules have been shown to exhibit stage-specific activity, particularly along the fibrosis-to-cirrhosis axis, indicating that regulatory interactions are not static but context-dependent. In this framework, MIR100HG expression and function may also vary across different stages of HCC, suggesting that its contribution to hypoxia-associated transcriptional responses is likely stage-dependent (REFF).

A major contribution of this study is the identification of the HIF-1α-mediated transcriptional regulation of MIR100HG. Bioinformatic analysis revealed four potential hypoxia response elements in the MIR100HG promoter, and ChIP experiments confirmed HIF-1α binding to multiple promoter regions, particularly those corresponding to the ChIP3 and ChIP5 fragments of the promoter. Notably, the integration of promoter deletion assays, ChIP-qPCR, and EMSA analyses provides converging evidence that MIR100HG is a direct transcriptional target of HIF-1α under hypoxic conditions. This is the first evidence showing that MIR100HG is a direct transcriptional target of HIF-1α in hypoxic conditions. Luciferase assays further supported these findings by demonstrating a significant increase in promoter activity under hypoxia, especially in the − 815/+231 fragment, suggesting that functional HRE sequences within this region mediate hypoxia-induced transcription. Previous studies have identified several transcription factors involved in the regulation of MIR100HG expression, including ELK-1 and SMAD2/3, while additional regulatory mechanisms have been suggested in cancer cells (Lu et al. 2017b). Later, in 2018, Ottaivani et al. showed that TGF-β-induced MIR100HG is upregulated in pancreatic cancer cells and that SMAD2 and 3 directly bind to the MIR100HG promoter and increase MIR100HG RNA when TGF-β was stimulated using a ChIP experiment (Ottaviani et al. 2018). Simultaneously, a ChIP experiment on osteosarcoma showed that the ELK-1 transcription factor binds to the MIR100HG promoter (Su et al. 2019). In this study, the determination of HRE binding sites to the MIR100HG promoter using a ChIP experiment was introduced to the literature for the first time under hypoxic conditions.

An apparent contrast exists between the previously reported oncogenic roles of MIR100HG and the inhibitory effects of hypoxia on proliferation and migration observed in this study. This difference likely reflects context-dependent function. While MIR100HG has been associated with proliferation under normoxic conditions, our findings suggest that under hypoxia it is linked to adaptive transcriptional responses accompanying cellular stress.

Notably, variation between epithelial-like and mesenchymal-like models further supports the context-dependent nature of MIR100HG activity. From a therapeutic perspective, targeting MIR100HG may therefore influence hypoxia-driven adaptation and tumor plasticity, representing a complementary strategy beyond direct inhibition of proliferation (Mukherjee et al. 2021).

Bioinformatic analysis of RNA-seq data from hypoxic and normoxic conditions showed that MIR100HG was significantly upregulated in Hep3B cells under hypoxia. The prominence of pathways related to the response to hypoxia, oxygen level-related biological processes, and metabolic adaptation in differential expression and enrichment analyses suggests that MIR100HG may be associated with and contribute to key components of the hypoxic response. In addition, enrichment of oxidative stress-related pathways, particularly KEAP1-NFE2L2 signaling, and proteostasis-associated mechanisms, including ubiquitin-mediated degradation and autophagy, indicates that MIR100HG may contribute to cellular adaptation beyond transcriptional activation alone. The literature has reported that MIR100HG is highly expressed in hepatocellular carcinoma and promotes cell proliferation and metastasis (Li et al., 2021). Furthermore, MIR100HG has been shown to have oncogenic functions in various cancers and regulate multiple signaling pathways (Ghafouri-Fard et al. 2023). Studies modulating the TGF-β signaling pathway have shown that MIR100HG may contribute to tumor progression (Papoutsoglou et al. 2021). In this context, the increased expression observed under hypoxia in our study suggests that MIR100HG contributes to tumor cell adaptation by interacting with the HIF-1α-mediated transcriptional networks. Our findings suggest that MIR100HG may be a novel regulatory component of hypoxia-associated transcriptional programs, and that this gene should be evaluated as a potential therapeutic target for hepatocellular carcinoma. Collectively, these findings position MIR100HG as a potential contributor to hypoxia-driven transcriptional and metabolic reprogramming in HCC.

Our GSEA under hypoxia revealed that cells developed extensive adaptive changes in oxygen-sensitive signaling networks in parallel with increased MIR100HG expression. Significant enrichment of pathways, particularly those related to the KEAP1-NFE2L2-mediated oxidative stress response, supports the role of MIR100HG in the transcriptional adaptation to hypoxia. The literature has reported that MIR100HG promotes tumor progression by facilitating proliferation and metastasis in hepatocellular carcinoma (Li et al., 2021), and its oncogenic functions in different cancer types have been summarized (Ghafouri-Fard et al. 2023). Furthermore, MIR100HG has been shown to enhances TGF-β signaling, fostering autocrine loops in tumor cells (Papoutsoglou et al. 2021) and regulating the cell cycle G1/S transition (Sun et al. 2018). In our study, the enrichment of proteasome, ubiquitin-dependent degradation, and autophagy pathways was consistent with these previous findings, suggesting that MIR100HG may play a role in both metabolic adaptation and maintenance of protein homeostasis during hypoxia-induced cellular reprogramming. Furthermore, the suppression of cadherin binding and ECM-related functions is noteworthy in terms of the effects of hypoxia on invasion and migration. Taken together, these findings suggest that the induction of MIR100HG under hypoxic conditions can be interpreted not only as a change in gene expression but also as part of a multifaceted adaptive mechanism that integrates proteasome activation, the oxidative stress response, and hypoxia-specific signaling networks within the cell.

The findings of this study suggest that MIR100HG remodels gene expression in HCC cells under hypoxic conditions. Confirmation of the results obtained from RNA-Seq analysis using qRT-PCR demonstrated that PAGE1 and CTAG2 genes, in particular, are regulated through MIR100HG. Importantly, functional manipulation experiments revealed that MIR100HG exerts a bidirectional regulatory effect on these genes, supporting its role as an active transcriptional modulator rather than a passive downstream target. The significant increase in PAGE1 under hypoxia may be considered a mechanism that may alter immune recognition of cancer cells in HCC and their interaction with the tumor microenvironment. PAGE1 is considered a potential target for immunotherapy due to its cancer-associated antigenic properties (Ai et al. 2023; M. E. Chen et al. 1998). In contrast, the decrease in CTAG2 expression under hypoxia suggests that this gene may be subject to context-specific regulatory mechanisms in HCC cells. While Liu et al. (2019) reported increased CTAG2 expression in HCC tissues, our study demonstrated that hypoxia reverses this expression pattern (Liu et al. 2019). This discrepancy highlights the context-dependent nature of hypoxia-driven transcriptional regulation within the tumor microenvironment.

Functional analyses support the direct or indirect transcriptional control of these genes by MIR100HG, as overexpression of MIR100HG increases PAGE1 and CTAG2 levels, whereas suppression with shRNA reverses this increase. The literature has reported that MIR100HG contributes to tumor progression by interacting with TGF-β and HIF-1α-mediated signaling pathways (Ghafouri-Fard et al. 2023; Papoutsoglou et al. 2021). Our findings suggest that, in addition to this axis, MIR100HG may also play a novel regulatory role by modulating selected components of tumor-immune interactions and hypoxia adaptation processes, particularly via PAGE1 and CTAG2. (Fig. 7).

Taken together, this study provides a comprehensive analysis of MIR100HG expression, promoter activity, transcription factor interactions, downstream targets, and the functional consequences of hypoxia in hepatocellular carcinoma. However, it should be noted that the present study is primarily based on in vitro models, and further in vivo validation and clinical correlation analyses are required to fully establish the translational relevance of the MIR100HG-HIF-1α axis. In addition, the use of chemical hypoxia (CoCl₂), while effective for stabilizing HIF-1α and enabling controlled mechanistic analyses, may not fully recapitulate physiological hypoxic conditions and may introduce context-specific effects independent of oxygen deprivation. For the first time, MIR100HG was defined as a direct HIF-1α-regulated lncRNA that participates in hypoxia-induced transcriptional reprogramming and may contribute to cellular adaptation by modulating oxidative stress pathways, protein homeostasis, metabolic remodeling, and immune-related gene expression. These findings position MIR100HG as a context-dependent regulator of hypoxia response in HCC and highlight its potential as both a prognostic biomarker and a therapeutic target.

## Supplementary Information

Below is the link to the electronic supplementary material.

Supplementary Table 1 (DOCX 23.8 KB)

Supplementary Material 1 (JPG 327 KB)

High Resolution Image (TIFF 5.79 MB)

## Data Availability

No datasets were generated or analysed during the current study.

## Abbreviations

MIR100HG: MicroRNA-100 Host Gene
HIF-1α: Hypoxia-Inducible Factor 1-alpha
CoCl₂: Cobalt(II) chloride
qRT-PCR: Quantitative Reverse Transcription Polymerase Chain Reaction
EMSA: Electrophoretic Mobility Shift Assay
ChIP-qPCR: Chromatin Immunoprecipitation followed by Quantitative PCR

## References

[CR1] Affo S, Yu LX, Schwabe RF (2016) The Role of Cancer-Associated Fibroblasts and Fibrosis in Liver Cancer. Annu Rev Pathol 12:153. 10.1146/ANNUREV-PATHOL-052016-100322 27959632 10.1146/annurev-pathol-052016-100322 PMC5720358

[CR2] Ai H, Yang H, Li L, Ma J, Liu K, Li Z (2023) Cancer/testis antigens: promising immunotherapy targets for digestive tract cancers. Front Immunol 14:1190883. 10.3389/fimmu.2023.1190883 37398650 10.3389/fimmu.2023.1190883 PMC10311965

[CR4] Alper M, Sav FN, Keleş Y, Eroğlu KP, Keskin SD, Köçkar F (2025) STAT-3, ELK-1, and c- Jun contributes IL-6 mediated ADAMTS-8 upregulation in colorectal cancer. Mol Biol Rep 52(1):246. 10.1007/s11033-025-10342-4 39969607 10.1007/s11033-025-10342-4

[CR3] Alper M, Kalfa Y, Sav FN, Eroğlu KP, Köçkar F (2025) TNF-α-induced upregulation of ADAMTS-8 expression in SW480 cells: implications for intracellular signaling pathways and transcription factor activity. Cell Biochem Biophys. 10.1007/s12013-025-01911-2 41042458 10.1007/s12013-025-01911-2

[CR5] Altuntaş C, Alper M, Keleş Y, Sav FN, Köçkar F (2023) Hypoxic regulation of ADAMTS-2 and – 3 (a disintegrin and matrix metalloproteinase with thrombospondin motifs 2 and 3) procollagen N proteinases by HIF-1α in endothelial cells. Mol Cell Biochem 478(5):1151–1160. 10.1007/s11010-022-04549-3 36241950 10.1007/s11010-022-04549-3

[CR6] Bagheri M, Sharifi 1 M, Salehi1 M, Sharifi M (2019) The effect of inhibition of LncRNA MIR100HG on the proliferation of human promyelocytic leukemia cells. Int J Life Sci Pharma Res 9(3):L11–L21. 10.22376/IJPBS/LPR.2019.9.3.L11-21

[CR7] Balas MM, Johnson AM (2018) Exploring the mechanisms behind long noncoding RNAs and cancer. Non-coding RNA Res 3(3):108–117. 10.1016/J.NCRNA.2018.03.001 10.1016/j.ncrna.2018.03.001 PMC6114262 30175284

[CR8] Benjaminit Y, Hochberg Y (1995) Controlling the False Discovery Rate: A Practical and Powerful Approach to Multiple Testing. J Royal Stat Soc Ser B: Stat Methodol 57(1):289–300. 10.1111/j.2517-6161.1995.tb02031.x

[CR9] Bevilacqua V, Gioia U, Di Carlo V, Tortorelli AF, Colombo T, Bozzoni I, Laneve P, Caffarelli E (2015) Identification of linc-NeD125, a novel long non coding RNA that hosts miR-125b-1 and negatively controls proliferation of human neuroblastoma cells. RNA Biol 12(12):1323–1337. 10.1080/15476286.2015.1096488 26480000 10.1080/15476286.2015.1096488 PMC4829289

[CR10] Bolger AM, Lohse M, Usadel B (2014) Trimmomatic: a flexible trimmer for Illumina sequence data. Bioinformatics (Oxford, England) 30(15):2114–2120. 10.1093/bioinformatics/btu170 24695404 10.1093/bioinformatics/btu170 PMC4103590

[CR11] Braga EA, Fridman MV, Burdennyy AM, Loginov VI, Dmitriev AA, Pronina IV, Morozov SG (2023) Various LncRNA mechanisms in gene regulation involving miRNAs or RNA-binding proteins in non-small-cell lung cancer: main signaling pathways and networks. Int J Mol Sci 24(17):13617. 10.3390/IJMS241713617 37686426 10.3390/ijms241713617 PMC10487663

[CR12] Chen ME, Lin S-H, Chung LWK, Sikes RA (1998) Isolation and Characterization of PAGE-1 andGAGE-7. J Biol Chem 273(28):17618–17625. 10.1074/jbc.273.28.17618 9651357 10.1074/jbc.273.28.17618

[CR13] Chen S, Xu H, Hu F, Wang T (2020) Identification of Key Players Involved in CoCl2 Hypoxia Induced Pulmonary Artery Hypertension in vitro. Front Genet 11:232. 10.3389/fgene.2020.00232 32391042 10.3389/fgene.2020.00232 PMC7193018

[CR14] Chen Z, Han F, Du Y, Shi H, Zhou W (2023) Hypoxic microenvironment in cancer: molecular mechanisms and therapeutic interventions. Signal Transduct Target Therapy 8(1):70. 10.1038/s41392-023-01332-8 10.1038/s41392-023-01332-8 PMC9935926 36797231

[CR15] Choi I, Lammata S, Merchant N, Park D (2017) Role of Hypoxia-Inducible Factor (HIF) in Liver Cancer. In *Role of Transcription Factors in Gastrointestinal Malignancies* (pp. 465–478). Springer Singapore. 10.1007/978-981-10-6728-0_35

[CR16] Dobin A, Davis CA, Schlesinger F, Drenkow J, Zaleski C, Jha S, Batut P, Chaisson M, Gingeras TR (2013) STAR: ultrafast universal RNA-seq aligner. Bioinf (Oxford England) 29(1):15–21. 10.1093/bioinformatics/bts635 10.1093/bioinformatics/bts635 PMC3530905 23104886

[CR17] Farooqi AA, Attar R, Qureshi MZ, Fayyaz S, Sohail MI, Uteuliyev YS, Sadykov BN, Yelekenova A, Yaylim I, Alaaeddine N (2018) Interplay of long non-coding RNAs and TGF/SMAD signaling in different cancers. Cell Mol Biol 64(15):1–6. 10.14715/CMB/2017.64.15.1 30672446

[CR18] Ghafouri-Fard S, Harsij A, Farahzadi H, Hussen BM, Taheri M, Mokhtari M (2023) A concise review on the role of < scp>MIR100HG</scp > in human disorders. J Cell Mol Med 27(16):2278–2289. 10.1111/jcmm.17875 37487022 10.1111/jcmm.17875 PMC10424294

[CR19] Hacıoğlu N, Güngör T, Tokay E, Önder FC, Ay M, Köçkar F (2020) Synthesis and biological evaluation of 2,4,6-Trinitroaniline derivatives as potent antitumor agents. Monatsh Chem. 10.1007/s00706-020-02690-7

[CR20] Hacıoğlu N, Güngör T, Tokay E, Gülhan ÜG, Çelik A, Ay M, Köçkar F (2021) Prodrugs for Nitroreductase Based Cancer Therapy-5: Development of Trinitroaniline Prodrugs/Ssap-NtrB Combinations for Liver Cancer Using Intracellular and Extracellular Conditions. ChemistrySelect 6(25):6315–6323. 10.1002/slct.202101115

[CR21] Kaplan EL, Meier P (1958) Nonparametric Estimation from Incomplete Observations. J Am Stat Assoc 53(282):457–481. 10.1080/01621459.1958.10501452

[CR22] Li F, Sun X, Liu Q, Liu X, Zhang J (2021) Long noncoding RNA MIR100HG knockdown attenuates hepatocellular carcinoma progression by regulating MicroRNA-146b-5p/Chromobox 6. Gastroenterol Res Pract 2021:6832518. 10.1155/2021/6832518 34381502 10.1155/2021/6832518 PMC8352691

[CR23] Liao Y, Smyth GK, Shi W (2014) featureCounts: an efficient general purpose program for assigning sequence reads to genomic features. Bioinformatics 30(7):923–930. 10.1093/bioinformatics/btt656 24227677 10.1093/bioinformatics/btt656

[CR24] Lin D, Wu J (2015) Hypoxia inducible factor in hepatocellular carcinoma: A therapeutic target. World J Gastroenterol 21(42):12171. 10.3748/WJG.V21.I42.12171 26576101 10.3748/wjg.v21.i42.12171 PMC4641134

[CR25] Liu J, Yu Z, Sun M, Liu Q, Wei M, Gao H (2019) Identification of cancer/testis antigen2 gene as a potential hepatocellular carcinoma therapeutic target by hub gene screening with topological analysis. Oncol Lett. 10.3892/ol.2019.10811 31611988 10.3892/ol.2019.10811 PMC6781590

[CR26] Livak KJ, Schmittgen TD (2001) Analysis of relative gene expression data using real-time quantitative PCR and the 2-∆∆CT method. Methods 25(4):402–408. 10.1006/meth.2001.1262 11846609 10.1006/meth.2001.1262

[CR27] Love MI, Huber W, Anders S (2014) Moderated estimation of fold change and dispersion for RNA-seq data with DESeq2. Genome Biol 15(12):550. 10.1186/s13059-014-0550-8 25516281 10.1186/s13059-014-0550-8 PMC4302049

[CR28] Lu Y, Zhao X, Liu Q, Li C, Graves-Deal R, Cao Z, Singh B, Franklin JL, Wang J, Hu H, Wei T, Yang M, Yeatman TJ, Lee E, Saito-Diaz K, Hinger S, Patton JG, Chung CH, Emmrich S, Klusmann J-H, Fan D, Coffey RJ (2017) lncRNA MIR100HG-derived miR-100 and miR-125b mediate cetuximab resistance via Wnt/β-catenin signaling. Nat Med 23(11):1331–1341. 10.1038/nm.4424 29035371 10.1038/nm.4424 PMC5961502

[CR29] Ma Y, Luo T, Dong D, Wu X, Wang Y (2018) Characterization of long non-coding RNAs to reveal potential prognostic biomarkers in hepatocellular carcinoma. Gene 663:148–156. 10.1016/j.gene.2018.04.053 29684484 10.1016/j.gene.2018.04.053

[CR30] Moore RA, Schmitz D (2016) Regarding Quality Improvement Demands Quality Data. Anesth Analg 122(6):2065. 10.1213/ANE.0000000000001230 27195648 10.1213/ANE.0000000000001230

[CR31] Mukherjee S, Kar A, Khatun N, Datta P, Biswas A, Barik S (2021) Familiarity breeds strategy: in silico untangling of the molecular complexity on course of autoimmune liver disease-to-hepatocellular carcinoma transition predicts novel transcriptional signatures. Cells 10(8):1917. 10.3390/cells10081917 34440687 10.3390/cells10081917 PMC8394127

[CR32] Ottaviani S, Stebbing J, Frampton AE, Zagorac S, Krell J, De Giorgio A, Trabulo SM, Nguyen VTM, Magnani L, Feng H, Giovannetti E, Funel N, Gress TM, Jiao LR, Lombardo Y, Lemoine NR, Heeschen C, Castellano L (2018) TGF-β induces miR-100 and miR-125b but blocks let-7a through LIN28B controlling PDAC progression. Nat Commun 9(1):3962-X. 10.1038/S41467-018-03962-X 29748571 10.1038/s41467-018-03962-x PMC5945639

[CR33] Ou L, Chen Y, Shen A, He S, Mei J, Meng Z, Yuan H, Wang X, Zhu X, Zhang L, Xing Y, Li F, Wang S, Pang X, Liu Y, Ma C (2026) Epigenetically activated MIR100HG regulates UPF1-mediated oxidative stress to promote pulmonary vascular immune microenvironment remodeling. Free Radic Biol Med 244:210–228. 10.1016/j.freeradbiomed.2025.11.038 41352378 10.1016/j.freeradbiomed.2025.11.038

[CR34] Papoutsoglou P, Rodrigues-Junior DM, Morén A, Bergman A, Pontén F, Coulouarn C, Caja L, Heldin C-H, Moustakas A (2021) The noncoding MIR100HG RNA enhances the autocrine function of transforming growth factor β signaling. Oncogene 40(21):3748–3765. 10.1038/s41388-021-01803-8 33941855 10.1038/s41388-021-01803-8 PMC8154591

[CR35] Peng J, Zhu Y, Dong X, Mao X, Lou Y, Mu Y, Xue D, Zhou H (2019) Construction and analysis of lncRNA-associated ceRNA network identified potential prognostic biomarker in gastric cancer. Translational Cancer Res 8(4):1116–1128. 10.21037/TCR.2019.06.32 10.21037/tcr.2019.06.32 PMC8798625 35116854

[CR37] Semenza GL (2010) HIF-1: upstream and downstream of cancer metabolism. Curr Opin Genet Dev 20(1):51–56. 10.1016/j.gde.2009.10.009 19942427 10.1016/j.gde.2009.10.009 PMC2822127

[CR38] Shang C, Zhu W, Liu T, Wang W, Huang G, Huang J, Zhao P, Zhao Y, Yao S (2016) Characterization of long non-coding RNA expression profiles in lymph node metastasis of early-stage cervical cancer. Oncol Rep 35(6):3185–3197. 10.3892/or.2016.4715 27035672 10.3892/or.2016.4715 PMC4869942

[CR39] Spearman C (1904) The Proof and Measurement of Association between Two Things. Am J Psychol 15(1):72. 10.2307/1412159

[CR40] Su X, Teng J, Jin G, Li J, Zhao Z, Cao X, Guo Y, Guo M, Li X, Wu J, Wang C, Guo Z, Guo Q (2019) ELK1-induced upregulation of long non-coding RNA MIR100HG predicts poor prognosis and promotes the progression of osteosarcoma by epigenetically silencing LATS1 and LATS2. Biomed Pharmacother 109:788–797. 10.1016/j.biopha.2018.10.029 30551532 10.1016/j.biopha.2018.10.029

[CR41] Sun Q, Tripathi V, Yoon J-H, Singh DK, Hao Q, Min K-W, Davila S, Zealy RW, Li XL, Polycarpou-Schwarz M, Lehrmann E, Zhang Y, Becker KG, Freier SM, Zhu Y, Diederichs S, Prasanth SG, Lal A, Gorospe M, Prasanth KV (2018) MIR100 host gene-encoded lncRNAs regulate cell cycle by modulating the interaction between HuR and its target mRNAs. Nucleic Acids Res 46(19):10405–10416. 10.1093/nar/gky696 30102375 10.1093/nar/gky696 PMC6212728

[CR36] R: The R Project for Statistical Computing (n.d.) Retrieved March 3, 2026 from https://www.r-project.org/

[CR42] Tokay E (2021) Epidermal Growth Factor Mediates Up-Regulation of URGCP Oncogene in Human Hepatoma Cancer Cells. Mol Biol 55(4):618–623. 10.1134/S0026893321030134 10.31857/S0026898421040133 34432785

[CR43] Tokay E, Kockar F (2016a) Identification of intracellular pathways through which TGF-β1 upregulates URG-4/URGCP gene expression in hepatoma cells. Life Sci 144:121–128. 10.1016/j.lfs.2015.12.010 26657209 10.1016/j.lfs.2015.12.010

[CR44] Tokay E, Kockar F (2016b) SP1 is a transcriptional regulator of URG-4/URGCP gene in hepatocytes. Mol Cell Biochem 423(1–2):75–83. 10.1007/s11010-016-2826-7 27766531 10.1007/s11010-016-2826-7

[CR45] Tokay E, Sagkan RI, Kockar F (2021) TNF-α Induces URG-4/URGCP Gene Expression in Hepatoma Cells through Starvation Dependent Manner. Biochem Genet. 10.1007/s10528-020-09972-z 33034821 10.1007/s10528-020-09972-z

[CR46] Wang H, Li X, Li T, Wang L, Wu X, Liu J, Xu Y, Wei W (2019) Multiple roles of microRNA-146a in immune responses and hepatocellular carcinoma. Oncol Lett 18(5):5033. 10.3892/OL.2019.10862 31612014 10.3892/ol.2019.10862 PMC6781720

[CR47] Wu Y, Wang Z, Yu S, Liu D, Sun L (2022) LncmiRHG-MIR100HG: a new budding star in cancer. Front Oncol 12:997532. 10.3389/FONC.2022.997532/FULL 36212400 10.3389/fonc.2022.997532 PMC9544809

[CR48] Yu G, Wang L-G, Han Y, He Q-Y (2012) clusterProfiler: an R package for comparing biological themes among gene clusters. OMICS 16(5):284–287. 10.1089/omi.2011.0118 22455463 10.1089/omi.2011.0118 PMC3339379

