# Supplementary material for: Hypoxia-driven transcriptional activation of MIR100HG by HIF-1α contributes to adaptive gene regulation in hepatocellular carcinoma
Source: Funct Integr Genomics. 2026 Jun 15;26(1):139. doi: 10.1007/s10142-026-01899-9 (PMC13265603; doi:10.1007/s10142-026-01899-9)
Supplement: Supplementary file 1 — Supplementary Table 1 (DOCX 23.8 KB) [file 10142_2026_1899_MOESM1_ESM.docx]

| **Name** | **Location** | **Primer sequence (5’-3’)** | **Base Pair** |
| --- | --- | --- | --- |
| **Promotor primers** | | | |
| MIR100HG Forward 1 | - | 5’-ACTCGAGTTTCCCTGAGAAATTGCAGA -3’ | - |
| MIR100HG Forward 2 | - | 5’-ACTCGAGTTTCCCTGAGAAATTGCAGA -3’ | - |
| MIR100HG Forward 3 | - | 5’-ACTCGAGTTCTCCATAAGCAACATAGAC- 3’ | - |
| MIR100HG Forward 4 | - | 5’-ACTCGAGTTCCACTGGACTCTATTAAAC-3’ | - |
| MIR100HG Forward 5 | - | 5’-ACTCGAGAAGTTAACTCCATTTTATGGAC-3’ | - |
| MIR100HG Reverse | - | 5’-AAAGCTTCTTTGTTACTATTCCTCCTTAAT-3’ | - |
| **ChIP Primers** | | | |
| ChIP 1_forward | +51/-155 | 5’-CAACTCACACAGAGGCTGTTCTT-3’ | 206 bp |
| ChIP 1_reverse | +51/-155 | 5’-AACTTCTGCGCTTCCAAAATTCC-3’ | 206 bp |
| ChIP 2_forward | -260/-465 | 5’-CAGTATGTGTCCAGAGCTCTAGA-3’ | 205 bp |
| ChIP 2_reverse | -260/-465 | 5’-CTGCATGGCTTACGCGCTGCAT-3’ | 205 bp |
| ChIP 3_forward | -748/-941 | 5’-GATAGGTATCGAGTCTAGATCTG-3’ | 193 bp |
| ChIP 3_reverse | -748/-941 | 5’-ACGTATGGTTCAGTATAGAGCTG-3’ | 193 bp |
| ChIP 4_forward | -1046/-1229 | 5’-CAGGAGAAAACTGGTCATGATGT-3’ | 183 bp |
| ChIP 4_reverse | -1046/-1229 | 5’-ATGGCAGAATGAAATAATACGTCA-3’ | 183 bp |
| ChIP 5_forward | -4734/-4909 | 5’-ATTAGGCGAAGTAAAAACAGAGTGA-3’ | 175 bp |
| ChIP 5_reverse | -4734/-4909 | 5’-TTCTTCAGAGATCTGCTTACATTC-3’ | 175 bp |
| ChIP 6_forward | -56979/-57107 | 5’-GATGAACACCCAGTATTGTTATGA-3’ | 128 bp |
| ChIP 6_reverse | -56979/-57107 | 5’-GTAAGAAGCTTTTACATTAAGCATG-3’ | 128 bp |
| **Silencing Primers** | | | |
| M1_Forward | 422 Starter Site (M1) | 5' CCGG—AAGACTGAAGAGACTGCTATA—CTCGAG—TATAGCAGTCTCTTCAGTCAA—TTTTTG 3' | - |
| M1_Reverse | 422 Starter Site (M1) | 5' AATTCAAAAA—AAGACTGAAGAGACTGCTATA—CTCGAG—TATAGCAGTCTCTTCAGTCAA 3' | - |
| M2_ Forward | 968 Starter Site (M2) | 5' CCGG—AAATCACTGCTTCTAAAGAGT—CTCGAG—ACTCTTTAGAAGCAGTGAAAA—TTTTTG 3' | - |
| M2_Reverse | 968 Starter Site (M2) | 5' AATTCAAAAA—AAATCACTGCTTCTAAAGAGT—CTCGAG—ACTCTTTAGAAGCAGTGAAAA 3' | - |
| M3_ Forward | 1830 Starter Site (M3) | 5' CCGG—AAGTTTATCAGCATTTCAGAC—CTCGAG— GTCTGAAATGCTGATAAACTT—TTTTTG 3' | - |
| M3_Reverse | 1830 Starter Site (M3) | 5' AATTCAAAAA—AAGTTTATCAGCATTTCAGAC—CTCGAG—GTCTGAAATGCTGATAAACTT 3' | - |
| M4_ Forward | 2907 Starter Site (M4) | 5' CCGG—AAAGTGTGACTTTGTTGACAT—CTCGAG—ATGTCAACAAAGTCACACTTT—TTTTTG 3' | - |
| M4_Reverse | 2907 Starter Site (M4) | 5' AATTCAAAAA—AAAGTGTGACTTTGTTGACAT—CTCGAG— ATGTCAACAAAGTCACACTTT 3' | - |
| **Expression Primers** | | | |
| MIR100HG_exp_Forward | - | 5’ AACTTGGCTTCCTCGCTTCT 3’ | - |
| MIR100HG_exp_Reverse | - | 5’ TGGCTCATGAAAATCTAGATGT 3’ | - |
| HIF1_Forward | - | 5’-CCACCTATGACCTGCTTGGT-3’ | - |
| HIF1_Reverse | - | 5’-TGTCCTGTGGTGACTTGTCC-3’ | - |
| PAGE1_exp_For | - | 5’- AATATGGGTTTTCTAAGAAGATT-3’ | 282 bp |
| PAGE1_exp_Rev | - | 5’- TTTCATCTGCTCTTCATTTCGCA-3’ | 282 bp |
| CTAG1A_exp_For | - | 5’- GCTTCAGGGCTGAATGGATGCT-3’ | - |
| CTAG1A_exp_Rev | - | 5’- TGAGCCAAAAACACGGGCAGAAA-3’ | - |
| Hß2_Forward | - | 5’ -TTTCTGGCCTGGAGGCTATC- 3’ | - |
| Hß2_Reverse | - | 5’ -CATGTCTCGATCCCACTTAACT- 3’ | - |
| Rat GAPDH Forward | - | 5’-CTGGAGAAACCTGCCAAGTATG-3’ | - |
| Rat GAPDH Reverse | - | 5’- GGTGGAAGAATGGGAGTTGCT-3’ | - |
