# Supplementary figures and images for: Hypoxia-driven transcriptional activation of MIR100HG by HIF-1α contributes to adaptive gene regulation in hepatocellular carcinoma
Source: Funct Integr Genomics. 2026 Jun 15;26(1):139. doi: 10.1007/s10142-026-01899-9 (PMC13265603; doi:10.1007/s10142-026-01899-9)

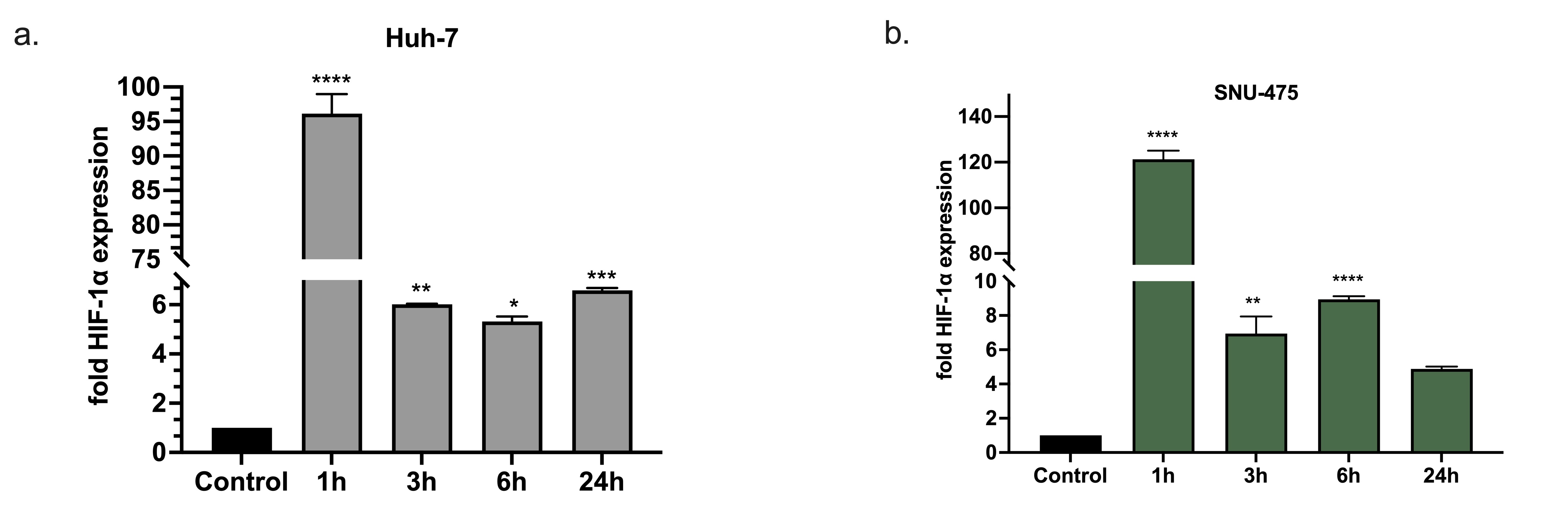

Supplement: Supplementary file 2 — Supplementary Material 1 (JPG 327 KB) [file 10142_2026_1899_MOESM2_ESM.jpg]

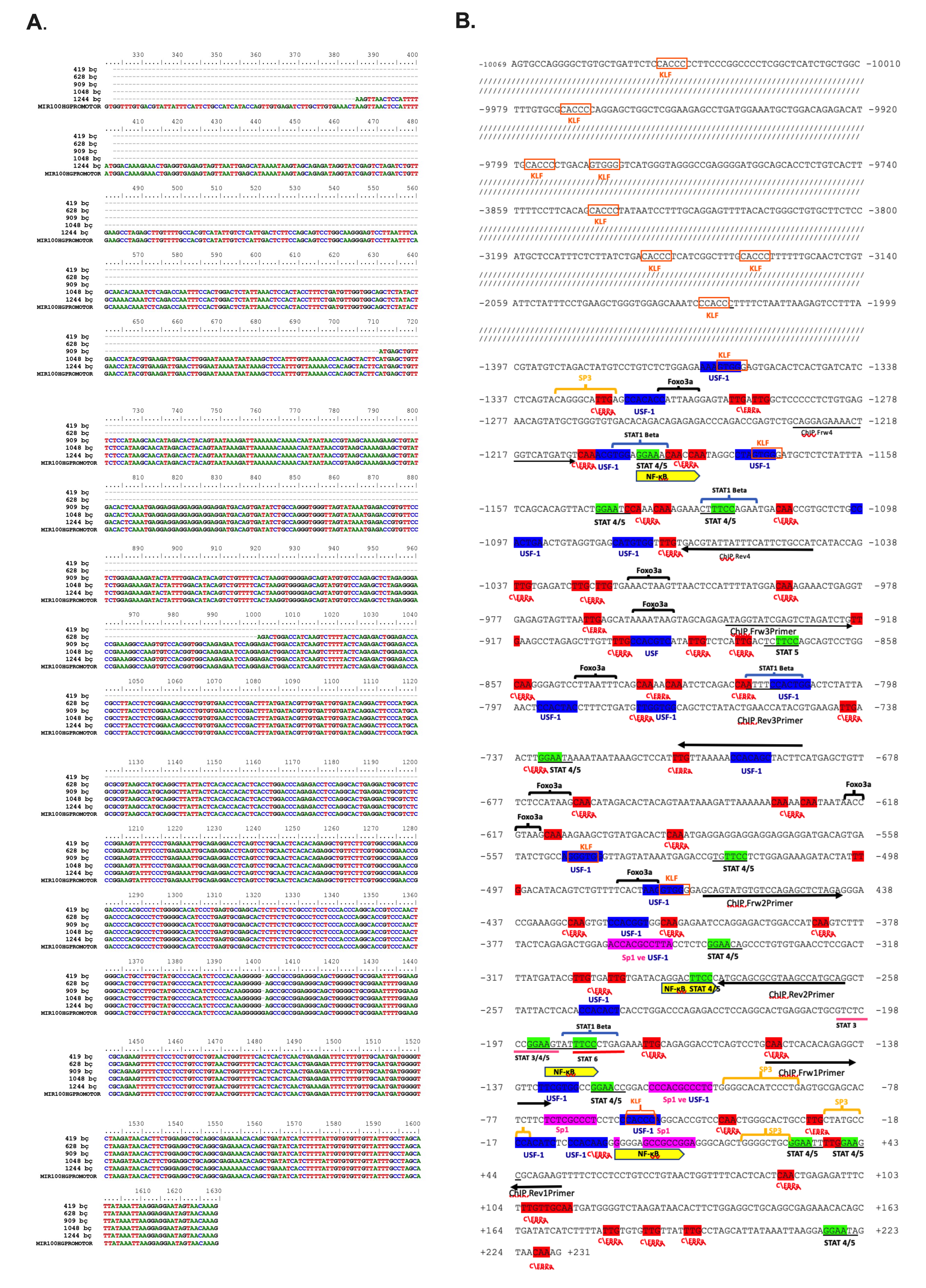

Supplement: Supplementary file 3 — High Resolution Image (TIFF 5.79 MB) [file 10142_2026_1899_MOESM3_ESM.tiff]
